# Supplementary material for: Chromatographic Data in Statistical Analysis of BBB Permeability Indices
Source: Membranes (Basel). 2023 Jun 26;13(7):623. doi: 10.3390/membranes13070623 (PMC10384010; doi:10.3390/membranes13070623)
Supplement: Supplementary file 1 [file membranes-13-00623-s001.zip › membranes-2455267-supplementary/Wanat Supplementary material/Wanat Table S1a Dataset of 181 APIs.pdf]

Table S1a. Dataset of 181 APIs

	API	CNS+/-	Ku,pp,brain	B1	B2 code	B2	B2>-0.9	B2>-0.52	PhCharg	pKa	logU/D	PB	NP/logD	NP/Sa	NP/MW	NP/V	NP	NP/PSA
1	**acebutolol**	0		0.0888	2	-0.8556	1	0	1	9.4	-2.2	0.26	1.73	0.07	0.13	0.14	0.45	0.52
2	**aceclofenac**			-0.4422	2	-0.6631	1	0	-1	2.6	-4.6	0.99	2.02	0.20	0.25	0.31	0.89	1.19
3	**acenocumarol**	1		-1.0731	1	-1.2037	0	0	-1	4.5	-2.7	0.987	2.91	0.21	0.28	0.33	0.99	0.90
4	**acetazolamid**	1			1	-1.2936	0	0	-1	7.44	-0.24	0.98	-1.71	0.20	0.34	0.32	0.94	0.82
5	**acetylsalicylic acid**	1		-4.7543	3	-0.4706	1	1	-1	3.48	-3.72	0.995	-0.47	0.29	0.49	0.58	0.89	1.41
6	**aciclovir**			-1.7069	1	-1.2103	0	0	0	5.11	-2.09	0.21	-0.41	0.20	0.32	0.39	0.73	0.66
7	**allopurinnol**	1		-0.0024	3	-0.5066	1	1	0	9.2	-2		-0.94	0.43	0.65	0.82	0.89	1.34
8	**alprazolam**	1		0.2859	3	-0.0632	1	1	0	2.37	-4.83	0.8	0.34	0.29	0.28	0.32	0.85	2.25
9	**amiodarone**	1		0.5965	3	-0.1359	1	1	1	9.37	-2.17	0.96	0.08	0.11	0.09	0.12	0.55	1.41
10	**amitriptyline**	1	1.33		3	0.4952	1	1	1	9.18	-1.98	0.95	0.10	0.09	0.14	0.21	0.32	9.57
11	**amlodipine**	0		-0.0905	1	-1.0511	0	0	1	8.97	-1.77	0.975	0.22	0.09	0.14	0.15	0.56	0.56
12	**amoxilcillin**	0		-2.2260	1	-1.9852	0	0	0	2.44	-4.76	0.2	-0.28	0.14	0.19	0.22	0.68	0.43
13	**astemizol**	1		-0.3065	3	-0.0512	1	1	1	8.68	-1.48	0.967	0.15	0.10	0.14	0.15	0.62	1.63
14	**atenolol**	0		-0.9866	2	-0.8063	1	0	1	9.43	-2.23		-0.20	0.12	0.13	0.15	0.34	0.39
15	**atorvastatin**	0		0.3731	1	-1.2416	0	0	-1	4.29	-2.91	0.98	0.81	0.13	0.16	0.17	0.9	0.81
16	**atropine**	1		-0.3377	3	-0.2493	1	1	1	9.98	-2.78	0.18	-0.29	0.06	0.09	0.10	0.27	0.52
17	**azithromycin**	0		0.5099	1	-2.3343	0	0	2	8.59	-1.39	0.29	0.20				0.63	0.35
18	**betahistine**	1		-0.0505	3	0.1483	1	1	1	9.75	-2.55		-0.08	0.05	0.13	0.12	0.17	0.64
19	**betaxolol**	0		0.2910	3	-0.2645	1	1	1	9.43	-2.23	0.5	0.71	0.11	0.21	0.21	0.66	1.28
20	**bilastine**			-0.7029	2	-0.7082	1	0	0	6.59	-0.61	0.87	0.23	0.07	0.10	0.10	0.46	0.56
21	**biperiden**	1		0.5509	3	0.1715	1	1	1	9.26	-2.06	0.6	0.40	0.17	0.22	0.21	0.68	2.77
22	**bisoprolol**	0		-0.4245	3	-0.4122	1	1	1	9.42	-2.22	0.3	1.68	0.09	0.21	0.20	0.67	1.09
23	**bromazepam**	1		-0.2717	3	-0.3226	1	1	0	11.39	-4.19	0.7	0.53	0.33	0.28	0.37	0.87	1.65
24	**bromocriptine**	0		-0.9295	1	-1.3444	0	0	0	6.44	-0.76	0.93	0.19	0.16	0.14	0.17	0.95	0.81
25	**bupivacaine**	1			3	0.0296	1	1	1	8.13	-0.93	0.95	0.24	0.14	0.23	0.22	0.67	2.07
26	**buspirone**	1		-0.7123	2	-0.5672	1	1	1	7.72	-0.52	0.95	0.17	0.11	0.15	0.15	0.57	0.83
27	**caffeine**	1		-0.8141	3	-0.3092	1	1	0	0.52	-6.68	0.3	-6.31	0.24	0.42	0.49	0.82	1.53
28	**capecitabine**	1		-1.5667	1	-1.3840	0	0	0	5.41	-1.79	0.6	-1.26	0.17	0.26	0.30	0.92	0.76
29	**captopril**	1		0.1846	3	-0.3748	1	1	-1	3.59	-3.61	0.275	-0.24	0.21	0.35	0.40	0.77	1.28
30	**carbamazepine**	1	0.76	-4.8027	3	-0.1943	1	1	0	13.94	-6.74	0.76	0.36	0.33	0.40	0.43	0.95	2.04
31	**carbegoline**	1		-0.8778	2	-0.5999	1	1	2	9.41	-2.21	0.41	-3.58	0.11	0.16	0.17	0.68	0.95
32	**carvedilol**	0		-0.1711	2	-0.6642	1	0	1	8.24	-1.04	0.98	0.22	0.13	0.17	0.18	0.73	1.19
33	**cefuroxime**	0		-3.1232	1	-2.6380	0	0	-1	2.59	-4.61	0.5	0.33	0.23	0.23	0.26	0.97	0.49
34	**celecoxib**	1		0.2940	2	-0.7007	1	0	0	9.68	-2.48	0.97	0.23	0.19	0.26	0.33	0.98	1.27
35	**celiprolol**			0.0706	1	-0.9074	0	0	1	9.5	-2.3	0.275	2.05	0.07	0.12	0.13	0.45	0.51
36	**cephalexin**	0		-0.0601	3	0.0267	1	1	0	3.12	-4.08	0.14	-0.37	0.21	0.26	0.31	0.9	2.77
37	**cetrizine**	1		-0.3248	3	-0.3012	1	1	0	3.46	-3.74	0.93	-0.57	0.13	0.17	0.19	0.64	1.22
38	**chloramphenikol**	0		-1.6066	1	-1.2991	0	0	0	11.03	-3.83	0.55	0.97	0.22	0.31	0.41	0.99	0.84
39	**chloroquine**	1			3	0.0964	1	1	2	10.47	-3.27	0.55	0.09	0.03	0.06	0.05	0.17	0.60
40	**chlorpromazine**	1	3.41	0.2417	3	0.0385	1	1	1	9.41	-2.21	0.9	0.13	0.14	0.13	0.16	0.43	1.34
41	**chlortalidone**	1		-1.7012	1	-1.3389	0	0	0	9.57	-2.37	0.75	-1.30	0.25	0.30	0.38	0.96	0.81
42	**cimetidine**	0		-1.6407	1	-1.2800	0	0	1	7.07	-0.13	0.17	-2.56	0.22	0.25	0.28	0.64	0.59
43	**ciprofloxacin**			-0.1422	2	-0.6191	1	0	0	7.56	-0.36	0.3	-0.13	0.03	0.00	0.04	0.11	0.15
44	**cisapride**	1		-0.7925	2	-0.8298	1	0	1	7.47	-0.27	0.975	0.25	0.10	0.14	0.16	0.64	0.73
45	**citalopram**	1	1.3		3	-0.0332	1	1	1	9.57	-2.37	0.8	0.77	0.05	0.09	0.10	0.3	0.80
46	**clarithromycin**	0		-2.5681	1	-2.3796	0	0	1	8.16	-0.96	0.7	0.42				0.99	0.54
47	**clindamycin**	0		0.3807	1	-1.1450	0	0	1	8.73	-1.53	0.93	1.44				0.69	0.62
48	**clobazam**	1		-0.6696	3	-0.0972	1	1	-1	8.59	-1.39	0.85	0.60	0.24	0.32	0.37	0.95	2.36
49	**clomipramine**	1			3	0.4433	1	1	1	9.46	-2.26	0.98	0.09	0.06	0.10	0.11	0.33	4.94
50	**clonidine**	1		-0.3575	3	-0.0357	1	1	1	8.1	-0.9	0.3	-0.72	0.16	0.21	0.26	0.49	1.37
51	**clorazepate**	1		-0.9548	1	-1.0874	0	0	-1	5.3	-1.9	0.975	0.61	0.32	0.30	0.37	0.94	0.92
52	**clozapine**	1	3.83	0.3601	3	0.4952	1	1	1	7.33	-0.13	0.99	0.09	0.12	0.16	0.16	0.45	14.04
53	**colchicine**	1			2	-0.7824	1	0	0			0.39	0.86	0.14	0.20	0.22	0.79	0.93
54	**cyproheptadine**	1		-0.0936	3	0.1049	1	1	1	8.95	-1.75	0.975	0.18	0.12	0.17	0.17	0.45	1.54
55	**desloratidine**	1		-0.2192	3	0.1483	1	1	1	10.27	-3.07	0.845	0.09	0.10	0.12	0.13	0.36	1.40
56	**diazepam**	1	0.96	0.1130	3	0.0243	1	1	0	3.4	-3.8	0.98	0.33	0.34	0.33	0.38	0.95	2.89
57	**digoxin**	0	1.8	-2.4604	1	-2.7020	0	0	0	13.5	-6.3	0.25	0.94	0.10	0.10	0.11	0.8	0.38
58	**dihydroergotamine**	0	0.02	-0.1817	1	-1.3444	0	0	1	7.22	-0.02	0.93	0.35	0.14	0.15	0.17	0.72	0.63
59	**diltiazem**	1			3	-0.3983	1	1	1	8.94	-1.74	0.75	0.23	0.08	0.11	0.12	0.49	0.83
60	**diphenhydramin**	1		0.2721	3	0.3475	1	1	1	8.76	-1.56	0.985	0.18	0.09	0.16	0.16	0.42	2.61
61	**doxazosin**	1		0.4643	1	-1.4162	0	0	1	6.52	-0.68	0.98	1.33	0.16	0.20	0.23	0.8	0.66
62	**doxepin**	1	3.11		3	0.3475	1	1	1	9.4	-2.2	0.755	0.14	0.06	0.11	0.11	0.3	2.49
63	**doxycycline**	0		-2.8317	1	-2.3589	0	0	0	7.67	-0.47	0.9	-0.02	0.01	0.01	0.02	0.06	0.02
64	**drotaverine**	1		-0.8150	3	-0.2362	1	1	1	6.17	-1.03	0.875	0.10	0.10	0.15	0.16	0.61	1.21
65	**duloxetine**	1			3	0.2068	1	1	1	10.02	-2.82	0.9	0.26	0.07	0.11	0.11	0.32	1.55
66	**eletriptan**	1			3	-0.3037	1	1	1	10.35	-3.15	0.85	0.81	0.06	0.08	0.08	0.29	0.53
67	**enalapril**	0		-0.9313	1	-0.9880	0	0	-1	3.15	-4.05	0.55	-0.14	0.08	0.09	0.09	0.33	0.34
68	**eplerenone**	1		-0.7308	2	-0.7154	1	0	0			0.5	0.90	0.21	0.23	0.25	0.94	1.19
69	**escitalopram**	1		0.2712	3	-0.0332	1	1	1	9.57	-2.37	0.56	1.08	0.08	0.13	0.14	0.42	1.16
70	**estradiol benzoate**			-0.9743	1	-1.0674	0	0	0			0.95	0.39	0.20	0.26	0.27	0.97	1.62
71	**estrone**			-0.8728	1	-1.0168	0	0	0	10.25	-3.05	0.95	0.39	0.21	0.25	0.27	0.99	1.01
72	**ethambutol**	0			3	-0.4853	1	1	1	9.59	-2.39	0.25	-0.09	0.04	0.09	0.09	0.19	0.33
73	**ethanol**	1			3	0.2233	1	1	0									
74	**famotidine**	1		-3.5484	1	-3.2570	0	0	1	7.93	-0.73	0.175	-0.69	0.14	0.21	0.26	0.7	0.30
75	**fexofenadine**	0		-0.4807	2	-0.7490	1	0	0	6.93	-0.27	0.65	0.27	0.10	0.12	0.13	0.61	0.73
76	**fluconazole**	1		-0.7958	2	-0.6016	1	0	0	11.01	-3.81		1.74	0.25	0.28	0.35	0.87	1.21
77	**fluoxetine**	1	5.23	0.4324	3	0.2068	1	1	1	10.05	-2.85	0.945	0.44	0.14	0.22	0.25	0.68	3.15
78	**flupenthixol**	1		0.1794	3	-0.2852	1	1	1	7.05	-0.15	0.95	0.12	0.09	0.12	0.14	0.51	1.00
79	**fluvoxamine**	1			3	-0.3624	1	1	1	9.39	-2.19	0.785	0.30	0.06	0.11	0.12	0.34	0.56
80	**furosemide**	0		-1.8364	1	-1.5492	0	0	-1	3.04	-4.16	0.95	-7.67	0.22	0.30	0.38	0.92	0.70
81	**gabapentin**	1	0.44		3	-0.4661	1	1	0	7.5	-0.295	0.03	-0.69	0.38	0.53	0.54	0.91	1.44
82	**gentamycin**	0			1	-2.6487	0	0	5	9.77	-2.57	0.15	-0.12	0.20	0.20	0.23	0.96	0.47
83	**gliclazide**	1		-0.8582	2	-0.8432	1	0	-1	6.07	-1.13	0.94	-3.39	0.19	0.29	0.33	0.95	1.09
84	**haloperidol**	1	1.77	-0.2619	3	-0.1016	1	1	1	8.04	-0.84	0.92	0.31	0.12	0.18	0.20	0.66	1.59
85	**hydrochlorotiazide**	0			1	-1.3468	0	0	0	8.95	-1.75	0.679	-10.55	0.25	0.32	0.48	0.96	0.81
86	**hydrocortisone acetate**			-0.1171	3	-0.4200	1	1	0	12.42	-5.22	0.95	0.21	0.20	0.26	0.27	0.96	1.59
87	**hydroxyzine**	1		-0.2060	3	-0.0280	1	1	1	6.62	-0.58	0.93	0.28	0.10	0.15	0.16	0.55	1.53
88	**ibuprofen**	1		0.1691	3	-0.0498	1	1	-1	4.41	-2.79	0.95	1.16	0.37	0.45	0.38	0.93	2.40
89	**indomethacin**	1		-1.0196	3	-0.4706	1	1	-1	3.96	-3.24	0.97	-5.50	0.18	0.25	0.29	0.88	1.42
90	**ipratropium**	1		0.5099	3	-0.1975	1	1	1				-0.20	0.08	0.12	0.12	0.43	0.92
91	**isosorbide**	1		-0.1939	1	-0.9528	0	0	0	13.09	-5.89		-0.51	0.09	0.14	0.18	0.26	0.25
92	**itraconazole**	0			1	-1.0656	0	0	0	6.47	-0.73	0.998	0.23	0.11	0.14	0.16	0.96	0.96

93	**ketoprofen**	1		0.5281	3	-0.3229	1	1	-1	4.23	-2.97	0.99	-3.56	0.22	0.35	0.38	0.89	1.64
94	**ketorolac**	1			3	-0.4018	1	1	-1	4.29	-2.91	0.99	-0.97	0.24	0.36	0.38	0.92	1.53
95	**ketotifen**	1		-0.5400	3	-0.2298	1	1	1	8.84	-1.64	0.75	0.10	0.09	0.11	0.12	0.34	0.74
96	**lamotrigine**	1	4.86	-0.9937	1	-0.9044	1	0	0	5.39	-1.81	0.55	-4.68	0.28	0.35	0.46	0.89	0.99
97	**levetiracetam**	1			3	-0.4674	1	1	0			0.1	-1.37	0.30	0.54	0.57	0.92	1.45
98	**levocetrizine**	1		-0.1166	3	-0.3012	1	1	0	3.46	-3.74	0.915	-0.56	0.11	0.16	0.18	0.63	1.21
99	**levofloxacin**			-0.8519	2	-0.6261	1	0	-1	5.19	-2.01	0.31	0.26	0.04	0.05	0.06	0.17	0.23
100	**lincomycin**	0			1	-1.4687	0	0	1	8.78	-1.58	0.7	-1.90	0.14	0.22	0.23	0.91	0.72
101	**loperamide**	0		0.2526	3	-0.1535	1	1	1	7.76	-0.56	0.97	0.19	0.14	0.14	0.16	0.68	1.53
102	**loratadine**	1		-0.4434	3	-0.1319	1	1	0	4.27	-2.93	0.98	0.16	0.19	0.25	0.28	0.94	2.22
103	**lorazepam**	1		-0.2542	3	-0.4400	1	1	0	10.8	-3.6	0.87	0.38	0.36	0.29	0.37	0.94	1.52
104	**medazepam**	1		0.5602	3	0.2974	1	1		6.18	-1.02	0.99	0.21	0.33	0.28	0.37	0.91	6.03
105	**meloxicam**	0		-2.0534	1	-1.6325	0	0	-1	4.5	-2.7	0.99	-11.00	0.25	0.28	0.37	0.99	0.72
106	**mesalazine**	0			2	-0.7898	1	0	-1	1.9	-5.3	0.43	-0.36	0.38	0.61	0.73	0.94	1.11
107	**metformin**	1		0.1922	2	-0.8768	1	0	2	12.25	-5.05		-0.05	0.08	0.00	0.16	0.21	0.25
108	**methyl paraben**			-0.5512	3	-0.1975	1	1		8.31	-1.11		0.54	0.31	0.64	0.72	0.97	2.08
109	**methyldopa**	0		-1.6189	1	-1.1135	0	0	0	5.57	-1.63	0.2	-0.16	0.11	0.18	0.21	0.38	0.35
110	**metoclopramide**	1	0.4	-0.6683	2	-0.5344	1	0	1	9.08	-1.88		14.50	0.05	0.10	0.10	0.29	0.43
111	**metoprolol**	0		0.3776	3	-0.2645	1	1	1	9.43	-2.23		14.12	0.07	0.16	0.15	0.48	0.95
112	**mianserin**	1	3.22	0.1860	3	0.4433	1	1	0	8.26	-1.06	0.9	0.20	0.15	0.20	0.20	0.54	8.41
113	**midazolam**	1	1.08	0.2836	3	0.1430	1	1	0	6.03	-1.17	0.97	0.21	0.27	0.25	0.30	0.82	3.27
114	**minoxidil**	1			1	-0.9511	0	0	0	5.54	-1.66		0.66	0.14	0.21	0.22	0.43	0.45
115	**mirtazapine**	1	3.85	0.0286	3	0.2371	1	1	0	8.1	-0.9	0.85	0.20	0.11	0.15	0.15	0.39	1.99
116	**montelukast**	1		0.7668	2	-0.5797	1	0	-1	4.76	-2.44	0.99	0.18	0.13	0.16	0.17	0.95	1.35
117	**naproxen**	1		-0.0952	3	-0.1975	1	1	-1	4.84	-2.36	0.99	1.96	0.24	0.40	0.43	0.92	1.91
118	**nebivolol**	1		-0.0176	2	-0.5882	1	0	1	8.65	-1.45	0.98	0.31	0.15	0.18	0.21	0.75	1.06
119	**nitrendipine**	0			1	-1.2202	0	0	0	2.79	-4.41	0.99	0.28	0.18	0.28	0.33	0.97	0.88
120	**ofloxacin**	0			2	-0.6261	1	0	-1	5.19	-2.01	0.32	-0.37	0.05	0.07	0.08	0.24	0.31
121	**olanzapine**		2.13	-1.3427	2	-0.7984	1	0	1	7.33	-0.13	0.175	-0.19	0.05	0.09	0.09	0.28	0.34
122	**oxazepam**	1		-0.3317	3	-0.4400	1	1	0	10.94	-3.74	0.85	0.40	0.34	0.32	0.34	0.93	1.52
123	**oxcarbamazepine**	1			3	-0.4674	1	1	0	13.73	-6.53	0.4	0.73	0.30	0.36	0.40	0.91	1.42
124	**oxybutinin**	1		0.6315	3	-0.2493	1	1	1	8.24	-1.04	0.92	0.12	0.12	0.19	0.19	0.68	1.33
125	**PABA**			-0.9106	3	-0.4661	1	1		4.86	-2.34		-0.55	0.35	0.65	0.73	0.89	1.41
126	**pantoprazole**	0		-0.1483	2	-0.8343	1	0	0	8.33	-1.13	0.98	0.62	0.18	0.24	0.31	0.93	1.07
127	**paracetamol**	1	0.73	-0.7917	3	-0.2423	1	1	0	9.86	-2.66	0.25	2.74	0.31	0.62	0.67	0.93	1.90
128	**paroxetine**	1		0.0645	3	-0.0885	1	1	1	9.68	-2.48	0.94	0.46	0.13	0.17	0.18	0.55	1.41
129	**pefloxacin**	0			3	-0.4784	1	1	-1	0.16	-7.04	0.25	0.58	0.06	0.09	0.10	0.3	0.45
130	**pergolide**	1	3.19	-0.1690	3	-0.1623	1	1	1	7.98	-0.78	0.9	0.21	0.11	0.15	0.16	0.48	1.06
131	**perindopril**	0		-0.9678	1	-0.9880	0	0	-1	3.15	-4.05	0.15	-47.00	0.09	0.13	0.13	0.47	0.47
132	**phenytoin**	1	0.66	-0.6114	3	-0.3842	1	1	0	6.32	-0.88	0.9	0.39	0.29	0.38	0.43	0.97	1.67
133	**pindolol**	1		-0.0480	3	-0.3695	1	1	1	9.54	-2.34	0.4	2.79	0.12	0.21	0.22	0.53	0.91
134	**piroxicam**	0		-1.2965	1	-1.1807	0	0	-1	4.5	-2.7	0.99	-0.46	0.37	0.29	0.39	0.98	0.91
135	**prednizolone**	1		-0.9590	1	-0.9703	0	0	0	12.46	-5.26	0.9	0.63	0.24	0.27	0.30	0.94	0.99
136	**pregabalin**	1			3	-0.4661	1	1	0	7.77	-0.57		-0.55	0.21	0.48	0.46	0.76	1.20
137	**primidone**	1	0.38		3	-0.3842	1	1	0	12.26	-5.06	0.7	2.25	0.28	0.41	0.45	0.9	1.60
138	**progesterone**			-0.0754	3	-0.1975	1	1	0			0.975	0.15	0.21	0.25	0.27	0.96	2.06
139	**promazine**	1		0.1627	3	0.0385	1	1	1	9.43	-2.23	0.94	0.15	0.13	0.14	0.15	0.39	1.16
140	**promethazine**	1		-0.3617	3	0.0385	1	1	1	8.98	-1.78	0.93	0.13	0.10	0.15	0.16	0.43	1.32
141	**propafenone**	0		0.4217	3	-0.3900	1	1	1	9.31	-2.11	0.97	0.28	0.11	0.17	0.17	0.57	1.01
142	**propranolol**	1		-0.0495	3	-0.1168	1	1	1	9.5	-2.3	0.9	0.43	0.17	0.21	0.21	0.54	1.21
143	**propylthiouracil**	1			3	-0.1111	1	1	0	7.63	-0.43	0.82	0.80	0.28	0.55	0.62	0.94	2.29
144	**pseudoephedrine**	1		0.2682	3	0.0308	1	1	1	9.38	-2.18		-0.99	0.27	0.54	0.52	0.89	2.76
145	**quetiapine**	1	1.26	-0.5186	2	-0.6306	1	0	1	6.74	-0.46	0.83	0.44	0.13	0.18	0.19	0.68	0.92
146	**quinapril**	0		0.3959	1	-0.9880	0	0	-1	3.39	-3.81	0.97	0.52	0.07	0.10	0.11	0.44	0.46
147	**quinine**	1			3	-0.1824	1	1	1	9.28	-2.08	0.7	0.19	0.07	0.10	0.10	0.3	0.64
148	**ranitidine**	0		-1.6914	1	-1.2380	0	0	1	8.35	-1.15		1.26	0.04	0.08	0.08	0.24	0.22
149	**rifampicin**	0			1	-2.9754	0	0	1	4.96	-2.24	0.89	-1.59		0.09		0.73	0.35
150	**rimantadine**	1			3	0.1307	1	1	1	10.76	-3.56	0.4	11.36	0.38	0.51	0.48	0.92	3.54
151	**risperidone**	1	0.21	-0.6820	3	-0.4440	1	1	1	8.07	-0.87	0.88	0.13	0.06	0.07	0.08	0.29	0.46
152	**rizatriptan**	1	0.04	-0.7769	3	-0.3988	1	1	1	9.49	-2.29	0.14	0.07	0.05	0.06	0.07	0.2	0.33
153	**rosuvastatin**	0		0.3184	1	-1.7077	0	0	-1	4.25	-2.95	0.88	-0.34	0.13	0.19	0.22	0.9	0.63
154	**roxitromicin**	0		0.5798	1	-2.9232	0	0	1	8.16	-0.96	0.96	0.31				0.91	0.42
155	**rupatadine**			-0.0427	3	0.0827	1	1	1	6.95	-0.25	0.985	0.07	0.08	0.10	0.11	0.42	1.48
156	**sertraline**	1	2.67	0.3944	3	0.3545	1	1	1	9.47	-2.27	0.98	0.19	0.13	0.17	0.20	0.53	4.41
157	**sildenafil**	1		-0.0844	1	-1.1991	0	0	0	6.03	-1.17	0.96	0.30	0.11	0.15	0.16	0.69	0.61
158	**simvastatin**	1		-0.2655	2	-0.6183	1	0	0	13.49	-6.29	0.95	0.04	0.03	0.05	0.05	0.19	0.23
159	**sotalol**	1		-1.2142	2	-0.8420	1	0	1	9.31	-2.11		-0.33	0.09	0.18	0.19	0.48	0.52
160	**spironolactone**	1		-0.6238	2	-0.8248	1	0	0			0.9	0.31	0.20	0.24	0.26	0.98	1.11
161	**sulpiride**	1		-1.4177	1	-1.2148	0	0	1	8.97	-1.77	0.4	-0.24	0.05	0.08	0.09	0.27	0.24
162	**telmisartan**	1		0.3287	3	-0.4623	1	1	-1	3.86	-3.34	0.995	0.19	0.14	0.18	0.19	0.9	1.42
163	**temazepam**	1	0.8	-0.1651	3	-0.2994	1	1	0	11.66	-4.46	0.96	0.44	0.33	0.31	0.37	0.94	1.77
164	**theophylline**	1		-1.0858	2	-0.5618	1	0	0	8.6	-1.4	0.4	-4.30	0.29	0.48	0.57	0.86	1.25
165	**thioridazine**	1	3.5	-0.0704	3	-0.3663	1	0	1	9.84	-2.64	0.95	0.12	0.10	0.13	0.13	0.47	0.75
166	**timolol**	0			2	-0.7288	1	0	1	9.35	-2.15	0.1	-0.24	0.07	0.11	0.12	0.34	0.40
167	**tinidazole**	1			1	-1.0175	0	0	0	2.3	-4.9	0.12	-3.30	0.20	0.36	0.45	0.89	0.90
168	**tolterodine**	1		0.6315	3	0.1715	1	1	1	10.68	-3.48	0.963	0.03	0.02	0.03	0.03	0.1	0.34
169	**tramadol**	1			3	0.0238	1	1	1	9.61	-2.41	0.2	0.86		0.12		0.31	0.95
170	**trazodone**	1	1.54	-0.0628	3	-0.1312	1	1	1	7.52	-0.32	0.92	0.45	0.14	0.19	0.21	0.71	1.69
171	**trimethoprim**	1			1	-1.1412	0	0	1	6.9	-0.3	0.44	0.98	0.14	0.20	0.22	0.57	0.56
172	**tropicamid**	1		0.1375	3	-0.3079	1	1	0	5.32	-1.88	0.45	0.74	0.20	0.30	0.31	0.85	1.61
173	**valproic acid**	1		0.5357	3	-0.0498	1	1	-1	4.82	-2.38	0.85	4.31	0.18	0.48	0.12	0.69	1.80
174	**valsartan**	0		-0.9299	1	-1.2461	0	0	-1	3.56	-3.64	0.955	17.80	0.16	0.21	0.23	0.89	0.81
175	**venlafaxine**	1	1.91	0.3579	3	0.0238	1	1	1	9.27	-2.07	0.27	0.34	0.08	0.13	0.13	0.36	1.13
176	**verapamil**	1		-0.7208	3	-0.4762	1	1	1	8.97	-1.77	0.9	0.23	0.07	0.12	0.12	0.54	0.82
177	**warfarin**	1			3	-0.4706	1	1	-1	4.5	-2.7	0.99	-1.10	0.25	0.32	0.36	0.99	1.56
178	**zolmitriptane**	1		-0.8634	3	-0.3708	1	1	1	9.52	-2.32	0.25	-0.70	0.07	0.11	0.11	0.31	0.51
179	**zolpidem**	1		-0.2869	3	-0.0548	1	1	0	6.77	-0.43	0.92	0.28	0.26	0.28	0.29	0.86	2.34
180	**zopiclone**	0		-0.8679	1	-0.9212	0	0	0	6.7	-0.5	0.45	-0.02	0.29	0.25	0.25	0.96	1.05
181	**zuclopenthixol**	1		0.1247	3	-0.2852	1	1	1	7.03	-0.17	0.985	0.09	0.08	0.12	0.13	0.47	0.90
