# Supplementary material for: Chromatographic Data in Statistical Analysis of BBB Permeability Indices
Source: Membranes (Basel). 2023 Jun 26;13(7):623. doi: 10.3390/membranes13070623 (PMC10384010; doi:10.3390/membranes13070623)
Supplement: Supplementary file 1 [file membranes-13-00623-s001.zip › membranes-2455267-supplementary/Wanat Supplementary material/Wanat Table S1b Dataset of 181 APIs.pdf]

Table S1b. Dataset of 181 APIs

	API	RP/logD	RP/Sa	RP/MW	RP/V	RP	RP/PSA	NP/PB	RP/PB	NP/logP	RP/logP	MW/100	PSA/10	HD	HA	HA+D	DM	eH	eL×10	eH-eL	logD	Sa/100	V/100	logP	k(IAM)	logkIAM	logIAM/PSA	logIAM/PB	logIAM/logP
1	**acebutolol**	3.00	0.12	0.23	0.24	0.78	0.94	1.77	3.15	-1.39	-2.48	3.36	8.77	3	5	8	2.65	-9.19	-5.11	-8.68	0.26	6.33	3.29	-0.33	5.06	0.70	-1.24	1.29	-2.13
2	**aceclofenac**	2.11	0.21	0.26	0.33	0.93	1.23	0.91	0.94	0.25	0.26	3.54	7.56	2	5	7	1.93	-8.85	-2.68	-8.58	0.44	4.44	2.84	3.54	2.63	0.42	-1.46	0.42	-0.13
3	**acenocumarol**	2.74	0.20	0.26	0.31	0.93	0.84	0.99	0.93	0.37	0.34	3.53	10.94	1	7	8	8.94	-9.74	-14.71	-8.27	0.34	4.66	2.96	2.68	0.53	-0.27	-2.31	-0.27	-0.70
4	**acetazolamid**	-1.76	0.21	0.35	0.33	0.97	0.83	0.96	0.98	0.40	0.41	2.77	11.50	2	5	7	0.89	-9.06	0.69	-9.13	-0.55	4.69	2.90	2.36					
5	**acetylsalicylic acid**	-0.52	0.32	0.54	0.64	0.98	1.56	0.90	0.99	0.69	0.76	1.80	6.36	1	4	5	5.18	-9.78	-6.93	-9.08	-1.89	3.05	1.52	1.3	0.03	-1.52	-3.32	-1.51	-1.63
6	**aciclovir**	-0.56	0.28	0.44	0.53	0.99	0.89	3.45	4.67	-0.50	-0.68	2.25	10.98	4	8	12	5.69	-8.76	-4.89	-8.27	-1.76	3.59	1.86	-1.45	0.09	-1.04	-3.08	-0.36	
7	**allopurinnol**	-1.02	0.47	0.71	0.90	0.97	1.41			-0.95	-1.00	1.36	6.59	2	5	7	8.84	-8.77	-10.78	-7.69	-0.95	2.06	1.08	-0.93					
8	**alprazolam**	0.32	0.27	0.26	0.30	0.8	2.05	1.08	0.98	0.18	0.17	3.09	3.81	0	4	4	6.16	-9.51	-1.42	-9.36	2.5	2.94	2.69	4.68	3.14	0.50	-1.08	0.59	-0.17
9	**amiodarone**	0.05	0.06	0.05	0.07	0.33	0.80	0.63	0.35	0.20	0.11	6.45	4.27	0	3	3	5.30	-7.46	-33.48	-4.11	6.94	5.21	4.46	3.01	99.63	2.00	0.37	2.02	1.52
10	**amitriptyline**	0.21	0.19	0.30	0.43	0.67	19.44	0.33	0.66	0.70	1.43	2.22	0.32	0	1	1	6.42	-9.90	-17.01	-8.20	3.15	3.61	1.56	0.44					
11	**amlodipine**	0.24	0.10	0.15	0.17	0.61	0.68	0.57	0.70	-0.37	-0.45	4.09	9.99	2	5	7	2.88	-8.66	-3.35	-8.32	2.59	5.92	3.62	-1.51	98.90	2.00	0.00	2.01	-1.32
12	**amoxilcillin**	-0.41	0.20	0.27	0.32	0.99	0.62	3.40	4.90	-4.53	-6.53	3.65	15.83	5	8	13	4.32	-9.20	-3.31	-8.87	-2.43	4.88	3.07	-0.15	0.05	-1.32	-3.52	-0.62	8.80
13	**astemizol**	0.16	0.11	0.14	0.15	0.65	1.66	0.63	0.64	0.86	0.87	4.59	3.74	1	5	6	1.71	-7.83	-3.07	-7.52	4.08	6.02	4.22	0.71	26.27	1.42	-0.15	1.43	1.57
14	**atenolol**	-0.33	0.20	0.22	0.25	0.58	0.66	3.00	5.09	0.40	0.67	2.66	8.46	4	5	9	6.13	-9.33	2.61	-9.59	-1.74	2.95	2.34	0.83	0.79	-0.10	-2.03		-0.02
15	**atorvastatin**	0.72	0.12	0.14	0.15	0.8	0.80	0.92	0.91	0.58	0.58	5.63	11.18	4	5	9	2.22	-9.00	-1.40	-8.86	1.11	6.75	5.21	1.54	8.28	0.92	-1.13	0.93	0.73
16	**atropine**	-0.78	0.17	0.25	0.26	0.73	1.45	1.44	4.00	0.15	0.42	2.89	4.98	1	4	5	2.48	-9.42	0.41	-9.46	-0.94	4.24	2.79	1.71	3.91	0.59	-1.10	1.34	0.36
17	**azithromycin**	0.08				0.24	0.13	2.17	0.83	0.26	0.10		18.01	5	13	18				0.00	3.18			2.44	0.06	-1.20	-3.46	-0.66	-1.59
18	**betahistine**	-0.14	0.09	0.23	0.21	0.31	1.32	4.00	8.25	0.14	0.28	1.36	2.49	1	2	3	2.91	-9.24	-0.60	-9.18	-2.18	3.27	1.44	1.18	0.90	-0.05	-1.44		-0.12
19	**betaxolol**	0.29	0.05	0.09	0.09	0.27	0.49	1.30	0.50	0.65	0.25	3.07	5.07	2	4	6	1.20	-8.98	4.43	-9.42	0.93	5.89	3.15	1	15.60	1.19	-0.51	1.49	1.19
20	**bilastine**	0.39	0.11	0.16	0.17	0.76	0.92	0.51	0.83	0.21	0.34	4.64	7.85	2	6	8	2.69	-8.82	-1.22	-8.70	1.97	6.65	4.54	2.1	0.55	-0.26	-2.15	-0.20	-0.58
21	**biperiden**	0.52	0.21	0.28	0.27	0.87	3.62	1.08	1.42	0.24	0.31	3.11	2.35	1	2	3	2.30	-9.16	3.22	-9.48	1.68	4.11	3.20	2.71	41.47	1.62	0.25	1.84	1.18
22	**bisoprolol**	1.43	0.08	0.18	0.17	0.57	0.93	2.18	1.87	0.31	0.26	3.25	6.00	2	5	7	1.93	-9.09	3.27	-9.42	0.4	7.05	3.31	2.13	6.74	0.83	-0.95	1.35	0.50
23	**bromazepam**	0.50	0.31	0.26	0.35	0.83	1.49	1.28	1.16	0.35	0.31	3.16	5.44	1	4	5	2.87	-9.13	-1.07	-9.02	1.65	2.67	2.36	2.59	2.21	0.34	-1.39	0.50	-0.07
24	**bromocriptine**	0.14	0.11	0.10	0.12	0.69	0.59	1.03	0.75	0.21	0.16	6.69	11.82	3	10	13	4.25	-8.92	-9.58	-7.96	5.07	6.06	5.64	4.48	12.69	1.10	-0.97	1.13	0.45
25	**bupivacaine**	0.26	0.15	0.26	0.25	0.74	2.32	0.71	0.79	0.18	0.20	2.88	3.23	1	2	3	3.02	-9.03	3.37	-9.37	2.8	4.78	3.02	3.82					
26	**buspirone**	0.21	0.13	0.18	0.19	0.7	0.99	0.61	0.73	0.49	0.58	3.86	6.96	0	7	7	4.32	-8.74	-1.56	-8.58	3.35	5.19	3.71	1.18	7.00	0.84	-1.00	0.87	0.77
27	**caffeine**	-7.00	0.27	0.47	0.54	0.91	1.68	2.73	3.00	-0.77	-0.85	1.94	5.35	0	6	6	3.92	-9.00	-5.36	-8.47	-0.13	3.41	1.68	-1.06	0.18	-0.75	-2.48	-0.23	0.71
28	**capecitabine**	-1.23	0.17	0.25	0.30	0.9	0.74	1.53	1.48	1.74	1.68	3.59	12.07	3	9	12	4.97	-9.72	-13.37	-8.38	-0.73	5.37	3.04	0.53	1.11	0.04	-2.04	0.27	0.32
29	**captopril**	-0.31	0.27	0.46	0.52	0.99	1.72	2.69	3.60	2.47	3.30	2.17	5.76	2	3	5	1.87	-9.35	1.05	-9.45	-3.15	3.63	1.92	0.3	0.01	-2.19	-3.95	-1.62	-1.66
30	**carbamazepine**	0.31	0.29	0.35	0.38	0.83	1.81	1.24	1.11	0.45	0.40	2.36	4.63	2	3	5	3.25	-9.05	-5.19	-8.53	2.67	2.85	2.19	2.1	1.50	0.18	-1.49	0.30	-0.15
31	**carbegoline**	-3.58	0.11	0.16	0.17	0.68	0.95	1.66	1.66	2.34	2.34	4.24	7.17	2	7	9	3.58	-8.39	-2.38	-8.15	-0.19	6.41	4.10	0.29	17.05	1.23	-0.62	1.62	1.77
32	**carvedilol**	0.18	0.11	0.14	0.15	0.59	0.81	0.92	0.62	-0.44	-0.30	4.20	7.57	3	5	8	3.15	-8.56	-2.09	-8.35	3.29	5.49	3.97	-2.04					
33	**cefuroxime**	0.30	0.21	0.21	0.23	0.88	0.44	1.96	1.74	-0.47	-0.42	4.24	19.91	12	4	16	2.73	-8.76	-13.17	-7.44	2.91	4.26	3.75	-2.08	1.69	0.23	-2.07	0.53	-0.11
34	**celecoxib**	0.18	0.15	0.20	0.25	0.75	0.96	1.02	0.77	0.97	0.74	3.81	7.80	1	3	4	4.09	-9.93	-14.21	-8.51	4.21	5.10	2.97	1.02	50.01	1.70	-0.19	1.71	1.69
35	**celiprolol**	3.41	0.11	0.21	0.21	0.75	0.87	1.67	2.87	-1.02	-1.76	3.65	9.09	3	5	8	2.01	-9.24	-4.56	-8.78	0.22	6.62	3.59	-0.45	5.87	0.77	-1.19	1.33	-1.71
36	**cephalexin**	-0.41	0.23	0.28	0.34	0.99	3.04	6.43	7.07	-0.69	-0.76	3.47	3.25	3	5	8	5.35	-9.59	-6.25	-8.97	-2.44	4.24	2.95	-1.31					
37	**cetrizine**	-0.73	0.17	0.22	0.25	0.83	1.55	0.69	0.88	0.31	0.39	3.75	5.30	1	5	6	3.56	-8.97	-2.20	-8.75	-1.13	5.01	3.37	2.11	1.05	0.02	-1.70	0.05	-0.30
38	**chloramphenikol**	0.87	0.20	0.28	0.36	0.89	0.77	1.76	1.62	-3.88	-3.56	3.23	11.54	3	7	10	-9.08	-10.34	-13.37	-9.01	1.02	4.50	2.44	-0.25	1.08	0.03	-2.03	0.29	-0.13
39	**chloroquine**	0.21	0.07	0.13	0.13	0.4	1.07	0.31	0.55	0.05	0.10	3.05	2.82	1	3	4	4.40	-8.60	-5.71	-8.03	1.87	5.94	3.11	3.11					
40	**chlorpromazine**	0.15	0.16	0.15	0.19	0.49	1.57	0.47	0.56	0.11	0.13	3.19	3.18	0	2	2	7.13	-6.83	0.74	-6.90	3.26	3.04	2.62	3.77	58.85	1.77	0.27	1.82	1.19
41	**chlortalidone**	-1.23	0.24	0.29	0.36	0.91	0.78	1.27	1.23	-1.52	-1.46	3.19	11.79	4	6	10	4.04	-9.45	-7.73	-8.68	-0.74	3.79	2.51	-0.63	1.32	0.12	-1.95	0.25	-0.19
42	**cimetidine**	-3.52	0.30	0.35	0.39	0.88	0.76	3.94	5.12	-1.14	-1.47	2.52	11.42	3	6	9	11.01	-8.75	-2.76	-8.48	-0.25	2.94	2.29	-0.59	0.26	-0.58	-2.64	0.19	0.98
43	**ciprofloxacin**	-0.45	0.09	0.00	0.13	0.38	0.29	0.37	0.70	-0.06	-0.11	331.35	7.29	2	6	8	6.93	-8.72	-7.85	-7.94	-0.85	4.25	2.84	-1.85	1.22	0.09	-1.77	0.61	-0.05
44	**cisapride**	0.25	0.10	0.14	0.16	0.65	0.72	0.65	0.64	0.28	0.28	4.66	8.61	3	7	10	1.68	-8.72	-1.86	-8.54	2.6	6.66	4.11	2.25	57.99	1.76	-0.17	1.77	1.41
45	**citalopram**	1.59	0.11	0.19	0.20	0.62	1.65	0.36	0.75	0.33	0.69	3.24	3.63	0	3	3	2.83	-7.98	8.83	-8.86	0.39	5.88	3.14	0.87					
46	**clarithromycin**	0.36				0.83	0.43	1.41	1.11	0.31	0.25		18.29	4	14	18				0.00	2.33			3.16	0.10	-1.02	-3.28	-0.86	-1.52
47	**clindamycin**	0.52				0.25	0.24	0.71	0.27	0.42	0.16		10.58	4	7	11				0.00	0.48			1.59	11.34	1.05	-0.97	1.09	0.85
48	**clobazam**	0.50	0.20	0.26	0.31	0.79	1.99	1.12	0.94	-0.68	-0.57	3.01	4.03	0	4	4	4.18	-9.21	-5.52	-8.66	1.59	4.02	2.58	-1.4	2.58	0.41	-1.19	0.48	
49	**clomipramine**	0.17	0.12	0.19	0.19	0.59	9.10	0.33	0.60	0.41	0.76	3.15	0.65	0	2	2	1.53	-8.49	1.56	-8.65	3.5	5.11	3.05	0.78					
50	**clonidine**	-1.07	0.24	0.32	0.39	0.73	1.87	1.67	2.27	1.79	2.43	2.30	3.64	2	3	5	3.51	-8.90	-4.25	-8.48	-0.68	3.10	1.86	0.28	2.83	0.45	-1.11	0.97	1.00
51	**clorazepate**	0.52	0.28	0.26	0.31	0.81	0.79	0.96	0.83	0.34	0.29	3.15	10.22	4	6	10	3.51	-9.12	-1.13	-9.01	1.55	2.91	2.57	2.75	4.88	0.69	-1.32	0.70	0.25
52	**clozapine**	0.12	0.15	0.20	0.20	0.57	17.59	0.46	0.58	0.26	0.32	2.87	0.32	0	1	1	1.13	-8.78	-2.69	-8.51	4.86	3.81	2.90	1.77	50.94	1.71	1.20	1.71	1.46
53	**colchicine**	0.99	0.17	0.23	0.25	0.91	1.10	1.97	2.33	-0.29	-0.34	3.99	8.31	1	6	7	5.95	-9.11	-5.91	-8.52	0.92	5.47	3.61	-2.65					

54	**cyproheptadine**	0.18	0.12	0.17	0.17	0.45	1.70	0.44	0.48	0.37	0.41	2.65	2.76	1	3	4	1.98	-8.63	2.03	-8.83	2.44	3.89	2.66	1.16	23.79	1.38	-0.06	1.39	1.31
55	**desloratidine**	0.14	0.15	0.19	0.20	0.58	2.33	0.41	0.69	5.00	8.29	3.11	2.49	1	2	3	3.04	-9.13	-2.94	-8.83	4.1	3.75	2.88	0.07	50.45	1.70	0.31	1.78	2.86
56	**diazepam**	0.26	0.28	0.27	0.31	0.77	2.36	0.96	0.79	0.31	0.26	2.85	3.27	0	3	3	3.57	-9.06	-1.00	-8.96	2.91	2.80	2.50	3.01	5.08	0.71	-0.81	0.71	0.23
57	**digoxin**	0.99	0.11	0.11	0.12	0.84	0.42	3.06	3.40	0.29	0.32	7.81	20.31	6	14	20	5.58	-8.45	-11.68	-7.28	0.85	7.66	7.05	2.67	3.69	0.57	-1.74	1.17	0.14
58	**dihydroergotamine**	0.38	0.15	0.16	0.18	0.78	0.66	0.81	0.84	-0.36	-0.37	4.78	11.82	3	6	9	3.90	-8.60	-1.05	-8.50	2.05	5.13	4.33	-2.11					
59	**diltiazem**	0.36	0.12	0.18	0.19	0.76	1.22	0.65	0.96	-0.44	-0.65	4.29	5.91	0	4	4	2.43	-8.71	-1.86	-8.52	2.1	6.17	3.96	-1.11					
60	**diphenhydramin**	0.25	0.12	0.23	0.22	0.58	4.33	0.33	0.55	0.16	0.26	2.55	1.25	0	2	2	0.52	-9.18	2.36	-9.41	2.29	4.92	2.65	2.09	3.66	0.56	-0.53	0.57	0.24
61	**doxazosin**	1.25	0.15	0.18	0.22	0.75	0.59	0.83	0.74	0.38	0.34	4.09	12.27	1	9	10	4.02	-8.67	-11.34	-7.54	0.6	4.93	3.46	2.14	0.22	-0.66	-2.75	-0.65	-0.99
62	**doxepin**	0.28	0.12	0.21	0.21	0.58	4.33	0.41	0.72	0.32	0.55	2.79	1.25	0	2	2	0.31	-9.11	-0.81	-9.03	2.08	4.69	2.81	0.98					
63	**doxycycline**	-0.02	0.02	0.02	0.02	0.08	0.05	0.05	0.10	-0.02	-0.05	4.44	18.16	7	10	17	11.15	-9.00	-11.51	-7.85	-3.32	4.43	3.68	-1.86	1.76	0.24	-2.01	0.29	-0.13
64	**drotaverine**	0.09	0.09	0.14	0.14	0.55	1.18	0.67	0.66	-0.39	-0.38	3.98	4.90	1	5	6	3.56	-8.36	-3.63	-7.99	6.12	6.39	3.89	-1.51	31.24	1.49	-0.20	1.55	-0.99
65	**duloxetine**	0.46	0.13	0.19	0.20	0.57	2.49	0.37	0.59	8.25	13.25	2.97	2.13	1	2	3	0.96	-9.01	-3.82	-8.63	1.24	4.56	2.84	0.04					
66	**eletriptan**	1.72	0.13	0.16	0.17	0.62	1.17	0.33	0.73	-0.80	-1.77	3.82	5.32	1	3	4	4.88	-8.50	-5.96	-7.90	0.36	4.69	3.57	-0.35					
67	**enalapril**	-0.26	0.14	0.16	0.17	0.6	0.60	0.59	1.05	0.14	0.25	3.75	9.59	2	7	9	3.46	-9.37	0.20	-9.38	-2.35	4.19	3.48	2.3	0.05	-1.29	-3.27	-1.03	-1.65
68	**eplerenone**	0.80	0.19	0.20	0.23	0.84	1.08	1.88	1.70	0.48	0.43	4.15	7.89	0	6	6	3.20	-10.28	-2.81	-10.00	1.05	4.43	3.73	1.96	1.54	0.19	-1.71	0.49	-0.10
69	**escitalopram**	1.41	0.10	0.17	0.18	0.55	1.43	0.75	0.93	0.48	0.60	3.24	3.63	0	3	3	2.57	-9.21	-8.44	-8.37	0.39	5.46	3.08	0.87	32.08	1.51	-0.05	1.76	1.57
70	**estradiol benzoate**	0.25	0.13	0.17	0.18	0.63	1.03	1.03	0.65	0.23	0.15	3.73	6.04	0	4	4	5.95	-10.11	-1.40	-9.97	2.51	4.77	3.58	4.2	6.92	0.84	-0.94	0.86	0.22
71	**estrone**	0.31	0.17	0.20	0.22	0.79	0.79	1.04	0.81	0.35	0.27	4.02	9.77	1	6	7	5.76	-10.17	-2.21	-9.95	2.53	4.73	3.67	2.86	29.40	1.47	-0.52	1.49	1.01
72	**ethambutol**	-0.44	0.20	0.48	0.44	0.97	1.47	0.84	3.80	0.72	3.28	2.04	6.45	4	4	8	1.11	-9.11	23.39	-11.45	-2.21	4.95	2.18	0.29					
73	**ethanol**											4.61	2.02	1	1	2					-0.19								
74	**famotidine**	-0.96	0.20	0.29	0.36	0.98	0.42	4.06	5.66	-0.64	-0.89	3.36	23.78	8	9	17	1.01	-8.55	-5.85	-7.97	-1.02	4.85	2.69	-1.11	0.26	-0.58	-2.96	0.17	0.53
75	**fexofenadine**	0.37	0.14	0.17	0.17	0.85	1.06	0.92	1.32	0.16	0.23	5.02	8.10	3	5	8	2.61	-8.98	-2.28	-8.75	2.3	6.27	4.87	3.81	1.08	0.03	-1.87	0.22	-0.55
76	**fluconazole**	1.80	0.26	0.29	0.36	0.9	1.24	7.57	7.74	1.04	1.06	3.06	7.18	1	7	8	0.52	-10.28	-0.83	-10.19	0.5	3.44	2.49	0.84	0.43	-0.37	-2.22		-0.29
77	**fluoxetine**	0.35	0.11	0.17	0.20	0.54	3.43	0.71	0.77	0.35	0.38	3.09	2.13	1	2	3	4.43	-9.41	-3.95	-9.02	1.56	4.99	2.71	1.93	66.64	1.82	0.50	1.85	1.54
78	**flupenthixol**	0.15	0.11	0.15	0.18	0.66	1.27	0.55	0.69	0.10	0.12	4.30	5.20	1	3	4	4.80	-7.61	-8.51	-6.76	4.42	5.85	3.65	5.33	51.21	1.71	-0.01	1.73	0.98
79	**fluvoxamine**	0.54	0.11	0.19	0.22	0.62	1.09	0.41	0.79	0.12	0.23	3.18	5.68	1	4	5	3.90	-9.69	-8.25	-8.87	1.15	5.89	2.79	2.69					
80	**furosemide**	-8.00	0.23	0.31	0.40	0.96	0.73	0.97	1.01	-3.83	-4.00	3.11	13.10	4	7	11	3.27	-8.92	-10.23	-7.90	-0.12	4.19	2.42	-0.24	0.28	-0.56	-2.67	-0.53	2.32
81	**gabapentin**	-0.50	0.28	0.39	0.39	0.66	1.04	30.33	22.00	0.95	0.69	1.71	6.33	2	3	5	1.56	-9.35	8.36	-10.19	-1.31	2.38	1.70	0.96					
82	**gentamycin**	-0.12	0.19	0.19	0.21	0.911	0.45	6.27	6.00	-0.27	-0.26	4.77	19.97	8	12	20	4.31	-9.34	18.62	-11.20	-7.81	4.71	4.25	-3.46					
83	**gliclazide**	-2.96	0.17	0.26	0.29	0.83	0.92	1.01	0.85	0.50	0.42	3.23	8.69	2	6	8	5.31	-9.38	-10.80	-8.30	-0.28	4.91	2.86	1.9	0.28	-0.55	-2.49	-0.53	-0.83
84	**haloperidol**	0.29	0.12	0.16	0.18	0.61	1.58	0.70	0.70	0.49	0.49	3.76	4.05	1	3	4	2.85	-9.22	-4.64	-8.75	2.11	5.29	3.37	1.31	23.74	1.38	-0.23	1.41	1.26
85	**hydrochlorotiazide**	-10.66	0.25	0.33	0.48	0.97	0.81	1.41	1.41	-0.30	-0.30	2.97	11.84	3	5	8	7.27	-9.64	-11.83	-8.46	-0.091	3.85	2.01	-3.24					
86	**hydrocortisone acetate**	0.18	0.17	0.22	0.23	0.82	1.36	1.01	0.86	0.23	0.20	3.73	6.04	0	4	4	4.23	-10.14	-0.86	-10.06	4.53	4.77	3.58	4.2	2.82	0.45	-1.33	0.47	-0.17
87	**hydroxyzine**	0.31	0.11	0.17	0.18	0.62	1.64	0.59	0.63	0.45	0.48	3.75	3.59	1	4	5	1.27	-8.81	-0.65	-8.75	2	5.48	3.54	1.23	13.82	1.14	-0.41	1.17	1.05
88	**ibuprofen**	1.04	0.33	0.40	0.34	0.83	2.17	0.94	0.85	0.23	0.21	2.06	3.73	1	2	3	1.91	-9.39	1.92	-9.58	0.8	2.52	2.42	3.83	0.86	-0.07	-1.64	-0.04	-0.65
89	**indomethacin**	-5.50	0.18	0.25	0.29	0.88	1.37	0.93	0.90	-0.63	-0.61	3.58	6.36	1	5	6	2.98	-8.69	-5.75	-8.12	-0.16	4.94	3.05	-1.43	1.08	0.03	-1.77	0.05	-0.02
90	**ipratropium**	-0.25	0.10	0.15	0.15	0.54	1.16	9.56	12.00	0.18	0.22	3.49	4.65	1	2	3	10.92	-12.36	-39.78	-8.39	-2.2	5.15	3.55	2.44	2.97	0.47	-1.19		0.09
91	**isosorbide**	-1.06	0.19	0.28	0.38	0.54	0.59	4.60	11.00	-0.11	-0.25	1.91	9.37	1	6	7	2.81	-11.01	-1.93	-10.82	-0.51	2.88	1.43	-2.19	0.21	-0.68	-2.65		
92	**itraconazole**	0.20	0.10	0.12	0.14	0.86	0.83	0.97	0.84	0.11	0.10	7.06	10.08	0	9	9	6.11	-8.42	-7.70	-7.65	4.26	8.43	6.15	8.68					
93	**ketoprofen**	-3.64	0.22	0.36	0.39	0.91	1.58	0.90	0.87	0.35	0.34	2.54	5.44	1	3	4	4.50	-9.96	-3.99	-9.56	-0.25	4.07	2.36	2.56	0.20	-0.71	-2.44	-0.70	-1.12
94	**ketorolac**	-1.01	0.25	0.37	0.40	0.96	1.62	0.92	0.97	0.51	0.54	2.59	5.93	1	3	4	2.14	-9.19	-3.63	-8.83	-0.95	3.87	2.39	1.79					
95	**ketotifen**	0.23	0.19	0.24	0.26	0.75	1.52	0.48	0.99	1.38	2.85	3.09	4.86	0	2	2	4.07	-9.08	-10.10	-8.07	3.28	3.89	2.87	0.26	22.04	1.34	-0.34	1.47	1.93
96	**lamotrigine**	-4.58	0.28	0.34	0.45	0.87	0.95	1.63	1.56	0.65	0.62	2.56	9.07	4	5	9	2.75	-8.22	-5.61	-7.66	-0.19	3.15	1.93	1.38	0.71	-0.15	-2.10	0.11	-0.29
97	**levetiracetam**	-1.37	0.30	0.54	0.57	0.92	1.45	9.20	9.20	-1.30	-1.30	1.70	6.34	1	2	3	5.71	-9.80	6.49	-10.45	-0.67	3.09	1.62	-0.71					
98	**levocetrizine**	-0.73	0.15	0.21	0.23	0.83	1.57	0.70	0.91	0.18	0.24	3.89	5.30	1	5	6	1.73	-9.00	-1.67	-8.83	-1.13	5.59	3.55	3.48	3.82	0.58	-1.14	0.62	0.04
99	**levofloxacin**	0.42	0.06	0.07	0.09	0.27	0.41	0.53	0.97	0.27	0.48	3.61	7.33	1	7	8	7.21	-8.72	-8.37	-7.88	0.65	4.75	3.06	0.62	0.90	-0.04	-1.91	0.46	0.16
100	**lincomycin**	-1.96	0.15	0.23	0.24	0.94	0.75	1.30	1.34	1.90	1.96	4.07	12.60	5	8	13	3.92	-8.50	0.04	-8.50	-0.48	6.35	3.89	0.48					
101	**loperamide**	0.21	0.15	0.16	0.17	0.75	1.76	0.69	0.79	0.13	0.15	4.77	4.38	1	4	5	4.65	-8.52	-0.23	-8.50	3.53	4.89	4.33	5.01					
102	**loratadine**	0.11	0.14	0.18	0.21	0.68	1.60	0.96	0.69	3.13	2.27	3.69	4.24	0	4	4	2.90	-9.02	-6.59	-8.36	5.94	4.93	3.31	0.3	11.76	1.07	-0.56	1.08	1.59
103	**lorazepam**	0.34	0.32	0.26	0.33	0.83	1.35	1.08	0.95	0.27	0.24	3.21	6.17	2	4	6	6.40	-7.77	-1.17	-7.65	2.47	2.58	2.53	3.42	9.04	0.96	-0.83	1.02	0.42
104	**medazepam**	0.15	0.24	0.20	0.27	0.66	4.17	0.95	0.66	0.22	0.15	3.30	1.56	0	2	2	4.08	-8.34	-0.59	-8.28	4.42	2.80	2.49	4.29	34.65	1.54	0.35	1.54	0.91
105	**meloxicam**	-10.56	0.24	0.27	0.35	0.95	0.70	0.99	0.96	-0.84	-0.82	3.51	13.62	2	7	9	5.35	-7.64	-12.59	-6.38	-0.09	4.03	2.71	-1.16	0.49	-0.31	-2.44	-0.31	0.27
106	**mesalazine**	-0.38	0.40	0.65	0.77	0.99	1.16	2.16	2.26	-0.53	-0.55	1.53	8.36	3	4	7	3.80	-8.18	-1.23	-8.06	-2.61	2.49	1.28	-1.76					
107	**metformin**	-0.18	0.30	0.01	0.61	0.78	0.75	220	670	0.63	1.91	129.60	8.90	4	5	9	1.58	-9.21	7.75	-9.99	-4.31	2.61	1.28	0.35	0.34	-0.47	-2.42		-0.02
108	**methyl paraben**	0.47	0.28	0.56	0.63	0.85	1.85			-97.00	-86.00	1.52	4.65	1	3	4	1.54	-9.53	-4.23	-9.11	1.81	3.08	1.35	-0.01	1.94	0.29	-1.38		-28.89

109	methyldopa	-0.36	0.25	0.40	0.46	0.85	0.83	1.83	4.30	-0.25	-0.59	2.11	10.38	5	5	10	1.56	-9.07	2.75	-9.34	-2.38	3.34	1.85	-1.46	0.10	-1.02	-3.03	-0.32	0.70
110	metoclopramide	32.50	0.12	0.22	0.23	0.65	0.95			0.23	0.50	3.00	6.76	3	5	8	3.80	-8.88	-2.89	-8.59	0.02	5.41	2.80	1.27	6.16	0.79	-1.04		0.69
111	metoprolol	8.24	0.04	0.09	0.09	0.28	0.49	4.00	2.08	0.31	0.16	3.09	5.07	2	4	6	1.53	-9.16	2.69	-9.43	0.034	7.00	3.22	1.57	4.32	0.64	-1.07		0.44
112	mianserin	0.20	0.15	0.21	0.21	0.55	8.49	0.61	0.61	0.58	0.59	2.64	0.65	0	2	2	0.56	-8.66	2.68	-8.93	2.76	3.65	2.64	0.94	21.72	1.34	0.52	1.38	1.36
113	midazolam	0.17	0.22	0.20	0.24	0.66	2.61	0.85	0.68	0.24	0.19	3.26	2.53	0	3	3	3.03	-9.24	-1.28	-9.11	3.92	3.04	2.76	3.41	5.69	0.76	-0.65	0.77	0.22
114	minoxidil	1.23	0.25	0.38	0.41	0.8	0.88			0.43	0.85	2.08	9.36	2	5	7	4.20	-8.07	0.29	-8.10	0.65	3.16	1.96	0.97					
115	mirtazapine	0.29	0.16	0.22	0.22	0.58	2.99	0.45	0.68	0.33	0.50	2.65	1.94	0	3	3	1.76	-8.79	0.19	-8.81	1.97	3.57	2.60	1.16	5.83	0.77	-0.52	0.84	0.70
116	montelukast	0.13	0.10	0.12	0.13	0.7	0.98	0.96	0.70	0.23	0.17	5.88	7.04	2	4	6	3.34	-7.46	-6.36	-6.83	5.19	7.27	5.47	4.13	99.10	2.00	0.15	2.00	1.38
117	naproxen	1.87	0.23	0.38	0.41	0.88	1.87	0.90	0.88	0.30	0.29	2.30	4.65	1	3	4	1.92	-9.10	-2.79	-8.82	0.47	3.89	2.15	2.99	0.31	-0.51	-2.18	-0.51	-0.99
118	nebivolol	0.17	0.08	0.10	0.11	0.4	0.56	0.77	0.41	-0.73	-0.39	4.05	7.10	3	5	8	2.46	-9.05	-1.07	-8.94	2.4	5.05	3.53	-1.03					
119	nitrendipine	0.25	0.16	0.25	0.29	0.86	0.78	0.98	0.87	-1.15	-1.02	3.46	11.05	1	5	6	6.92	-9.09	-10.94	-8.00	3.5	5.30	2.98	-0.84					
120	ofloxacin	-0.83	0.11	0.15	0.18	0.54	0.68	0.72	1.56	-0.09	-0.19	3.61	7.33	1	7	8	4.68	-8.72	-8.97	-7.82	-0.65	4.74	3.07	-2.61					
121	olanzapine	-0.36	0.09	0.16	0.18	0.53	0.59	1.63	2.86	-0.18	-0.32	3.28	8.41	1	6	7	7.55	-9.20	-7.85	-8.41	-1.48	6.19	3.01	-1.56	20.31	1.31	-0.62	2.06	-0.84
122	oxazepam	0.36	0.31	0.29	0.31	0.83	1.36	1.10	0.99	0.32	0.29	2.87	6.17	2	4	6	3.38	-9.09	-1.06	-8.98	2.32	2.72	2.72	2.91	4.78	0.68	-1.11	0.75	0.22
123	oxcarbamazepine	0.73	0.30	0.36	0.40	0.91	1.44	2.25	2.28	-2.20	-2.22	2.52	6.34	1	2	3	3.46	-9.31	-4.73	-8.84	1.25	3.02	2.26	-0.41					
124	oxybutinin	0.05	0.05	0.08	0.08	0.29	0.48	0.72	0.26	0.20	0.07	3.57	4.98	1	3	4	1.02	-9.18	-0.66	-9.11	5.53	5.82	3.61	3.24	49.68	1.70	0.00	1.73	1.19
125	PABA	-0.58	0.37	0.68	0.76	0.93	1.47			-1.21	-1.26	1.37	6.33	3	3	6	4.29	-8.50	-2.10	-8.29	-1.61	2.54	1.22	-0.74	0.01	-1.88	-3.69		2.55
126	pantoprazole	0.57	0.17	0.22	0.28	0.85	0.96	0.94	0.85	-0.49	-0.44	3.83	8.63	1	6	7	7.25	-9.09	-9.03	-8.19	1.5	5.08	3.01	-1.89	2.19	0.34	-1.60	0.35	-0.18
127	paracetamol	2.71	0.30	0.61	0.66	0.92	1.86	3.74	3.68	-0.71	-0.70	1.51	4.93	2	3	5	3.28	-8.58	0.73	-8.65	0.34	3.03	1.39	-1.32	0.32	-0.49	-2.19	0.11	0.37
128	paroxetine	0.50	0.15	0.18	0.20	0.6	1.69	0.60	0.71	-1.14	-1.37	3.31	3.97	1	4	5	2.12	-9.00	0.34	-9.04	1.19	4.12	2.99	-0.49	115.77	2.06	0.46	2.09	-4.21
129	pefloxacin	1.46	0.16	0.23	0.26	0.76	1.09	1.16	2.80	-0.19	-0.45	3.33	6.41	1	6	7	9.41	-9.01	-10.45	-7.97	0.52	4.66	2.91	-1.54					
130	pergolide	0.24	0.12	0.18	0.18	0.55	1.20	0.52	0.59	0.21	0.23	3.14	4.43	1	2	3	1.93	-8.35	1.51	-8.50	2.29	4.50	3.09	2.29	0.00				
131	perindopril	-57.00	0.10	0.15	0.16	0.57	0.60	3.03	3.87	0.22	0.28	3.68	9.59	2	7	9	6.26	-9.78	2.88	-10.07	-0.01	5.43	3.52	2.06	0.18	-0.76	-2.74	0.07	-1.07
132	phenytoin	0.35	0.26	0.34	0.39	0.87	1.48	1.08	0.96	1.33	1.18	2.52	5.82	2	4	6	2.98	-9.84	-2.94	-9.55	2.48	3.32	2.26	0.73	1.78	0.25	-1.52	0.30	0.39
133	pindolol	4.00	0.17	0.31	0.31	0.76	1.36	1.30	1.95	-0.42	-0.63	2.48	5.73	3	3	6	3.19	-8.47	0.63	-8.53	0.19	4.60	2.44	-1.23	6.09	0.78	-0.97	1.18	-0.64
134	piroxicam	-0.27	0.22	0.17	0.23	0.58	0.56	0.99	0.61	0.92	0.56	3.33	10.80	2	7	9	11.24	-8.63	2.05	-8.84	-2.14	2.66	2.50	1.07	0.16	-0.79	-2.82	-0.78	-0.82
135	prednizolone	0.58	0.22	0.25	0.28	0.87	0.93	1.04	0.98	0.47	0.44	3.46	9.48	3	5	8	3.68	-10.01	-4.70	-9.54	1.49	3.96	3.14	2.01	2.84	0.45	-1.52	0.50	0.15
136	pregabalin	-0.30	0.12	0.26	0.25	0.41	0.65			0.85	0.46	1.59	6.33	2	3	5	1.67	-9.53	9.29	-10.46	-1.38	3.54	1.65	0.89					
137	primidone	2.33	0.29	0.43	0.46	0.93	1.67	1.33	1.39	1.29	1.35	2.18	5.82	2	2	4	2.84	-9.78	-0.15	-9.77	0.4	3.19	2.02	0.72					
138	progesterone	0.11	0.16	0.19	0.20	0.71	1.46	0.98	0.70	0.31	0.22	3.77	4.65	1	3	4	5.88	-9.29	-2.60	-9.03	6.24	4.54	3.60	3.12	34.99	1.54	-0.12	1.55	1.05
139	promazine	0.19	0.17	0.18	0.20	0.51	1.60	0.39	0.54	0.11	0.16	2.84	3.18	0	2	2	2.86	-7.54	-3.93	-7.15	2.67	2.93	2.53	3.25	0.10	-1.02	-2.52	-0.99	-1.53
140	promethazine	0.15	0.11	0.17	0.18	0.49	1.48	0.45	0.51	-2.10	-2.35	2.84	3.18	0	2	2	3.40	-7.83	-0.65	-7.76	3.2	4.36	2.76	-0.2	14.96	1.17	-0.33	1.21	-5.87
141	propafenone	0.27	0.10	0.17	0.17	0.56	1.04	0.61	0.63	0.32	0.33	3.29	5.86	2	4	6	2.97	-8.77	-1.75	-8.59	2.06	5.36	3.28	1.86	66.74	1.82	0.06	1.84	1.55
142	propranolol	0.50	0.21	0.25	0.25	0.64	1.52	0.56	0.70	0.18	0.23	2.59	4.15	2	3	5	1.53	-8.36	-5.71	-7.79	1.27	3.09	2.56	2.8	28.09	1.45	-0.17	1.49	1.00
143	propylthiouracil	0.79	0.28	0.55	0.62	0.93	2.26	1.15	1.13	1.32	1.31	1.70	4.11	2	1	3	5.57	-9.20	-12.71	-7.93	1.18	3.32	1.51	0.71					
144	pseudoephedrine	-0.60	0.16	0.33	0.32	0.54	1.74	890	560	1.05	0.66	1.65	3.23	2	2	4	2.62	-9.25	5.04	-9.75	-0.9	3.30	1.71	0.85	2.21	0.34	-1.16		0.42
145	quetiapine	0.47	0.13	0.19	0.21	0.73	0.94	0.82	0.83	0.24	0.24	3.84	7.36	1	5	6	1.57	-8.68	-7.24	-7.95	1.55	5.42	3.55	2.84	17.02	1.23	-0.64	1.31	0.78
146	quinapril	0.67	0.09	0.13	0.14	0.57	0.58	0.45	0.58	0.26	0.33	4.39	9.59	2	5	7	5.13	-9.50	0.91	-9.59	0.85	6.10	4.10	1.69	1.22	0.09	-1.89	0.10	-0.14
147	quinine	0.42	0.16	0.21	0.22	0.66	1.40	0.41	0.91	0.24	0.53	3.14	4.56	1	4	5	1.73	-9.55	-0.14	-9.54	1.58	4.22	3.06	1.2					
148	ranitidine	4.26	0.13	0.26	0.28	0.81	0.72	1.60	5.33	-0.20	-0.68	3.14	11.16	2	7	9	8.02	-9.24	-7.19	-8.52	0.19	6.30	2.89	-1.18	0.67	-0.17	-2.22		0.15
149	rifampicin	-2.07		0.12		0.95	0.43	0.87	1.06			8.23	22.02	6	14	20					-0.46								
150	rimantadine	11.73	0.39	0.53	0.50	0.95	3.65	2.30	2.38	0.42	0.43	1.79	2.60	1	1	2	1.22	-9.33	29.54	-12.28	0.081	2.43	1.91	2.19					
151	risperidone	0.27	0.13	0.15	0.17	0.62	0.94	0.32	0.66	0.45	0.92	4.10	6.19	0	6	6	3.42	-8.93	-8.76	-8.06	2.27	4.91	3.73	0.63	4.83	0.68	-1.11	0.74	0.88
152	rizatriptan	0.27	0.17	0.23	0.25	0.72	1.30	1.39	5.50	-0.72	-2.85	3.12	5.91	1	4	5	1.76	-8.14	-6.90	-7.45	2.68	4.26	2.90	-0.27	2.53	0.40	-1.37	1.26	-1.49
153	rosuvastatin	-0.35	0.13	0.19	0.22	0.92	0.62	1.01	1.00	0.75	0.75	4.82	14.09	3	8	11	5.66	-9.19	-10.63	-8.13	-2.63	6.89	4.12	1.18	0.96	-0.02	-2.17	0.04	-0.09
154	roxitromicin	0.28				0.8	0.36	0.96	0.82	0.32	0.27		21.69	5	16	21				0.00	2.9			2.9					
155	rupatadine	0.09	0.11	0.13	0.14	0.55	1.79	0.44	0.53	0.26	0.32	4.15	2.90	0	3	3	1.61	-8.99	-6.43	-8.35	5.97	4.97	3.94	1.63	9.29	0.97	-0.49	0.97	0.76
156	sertraline	0.18	0.12	0.16	0.18	0.49	3.99	0.54	0.49	0.32	0.29	3.06	1.20	1	1	2	2.87	-9.19	-0.94	-9.09	2.77	4.21	2.72	1.68	42.89	1.63	0.55	1.64	1.41
157	sildenafil	0.34	0.12	0.16	0.18	0.77	0.70	0.70	0.79	-0.46	-0.52	4.75	10.91	1	8	9	5.84	-8.91	9.47	-9.86	2.27	6.47	4.20	-1.47	14.26	1.15	-0.88	1.17	-0.79
158	simvastatin	0.15	0.12	0.16	0.16	0.66	0.89	0.18	0.68	0.04	0.15	4.19	7.28	1	5	6	5.78	-9.10	2.25	-9.33	4.41	5.71	4.10	4.43	13.21	1.12	-0.74	1.14	0.47
159	sotalol	-0.59	0.16	0.31	0.34	0.85	0.98	45.50	85.00	-1.01	-1.89	2.72	8.68	3	5	8	5.15	-9.09	-5.13	-8.58	-1.45	5.19	2.48	-0.45	1.35	0.13	-1.81		-0.29
160	spironolactone	0.25	0.16	0.19	0.20	0.78	0.90	1.06	0.86	0.29	0.23	4.17	8.57	0	4	4	1.67	-9.76	-7.98	-8.96	3.12	4.94	3.82	3.33	13.38	1.13	-0.81	1.17	0.60
161	sulpiride	-0.76	0.17	0.25	0.28	0.85	0.78	0.65	2.15	0.54	1.79	3.41	11.01	3	7	10	3.65	-7.89	-16.74	-6.22	-1.12	5.08	3.02	0.48	1.66	0.22	-1.82	0.62	0.54
162	telmisartan	0.18	0.14	0.17	0.19	0.87	1.35	0.90	0.85	0.12	0.12	5.01	6.31	1	6	7	0.71	-8.56	-5.48	-8.02	4.76	6.39	4.69	7.39	4.68	0.67	-1.13	0.67	-0.20
163	temazepam	0.37	0.28	0.26	0.31	0.79	1.49	0.97	0.82	0.30	0.25	3.01	5.29	1	4	5	2.48	-9.17	-0.83	-9.09	2.15	2.84	2.53	3.15	6.74	0.83	-0.89	0.85	0.33

164	**theophylline**	-4.70	0.31	0.52	0.63	0.94	1.34	2.16	2.33	-0.66	-0.71	1.80	6.93	1	6	7	3.47	-9.10	-5.77	-8.53	-0.2	3.02	1.50	-1.31	0.12	-0.94	-2.78	-0.54	0.72
165	**thioridazine**	0.10	0.08	0.11	0.11	0.4	0.70	0.45	0.42	0.10	0.10	3.71	5.71	0	2	2	0.98	-7.92	-2.68	-7.65	3.94	4.89	3.50	4.18	47.61	1.68	-0.08	1.70	1.06
166	**timolol**	-0.70	0.20	0.31	0.34	0.97	1.22	3.20	9.70	0.26	0.80	3.16	7.97	2	7	9	3.06	-9.17	-11.24	-8.05	-1.39	4.95	2.88	1.21					
167	**tinidazole**	-3.48	0.21	0.38	0.47	0.94	0.95	7.33	7.75	29.33	31.00	2.47	9.78	0	5	5	2.34	-10.52	-12.82	-9.24	-0.27	4.41	2.00	0.03					
168	**tolterodine**	0.15	0.08	0.14	0.13	0.45	1.83	0.08	0.45	0.02	0.13	3.25	2.35	1	2	3	2.18	-8.95	2.20	-9.17	2.94	5.73	3.44	3.24	0.21	-0.68	-2.05	-0.66	-1.19
169	**tramadol**	1.75		0.24		0.63	1.96	1.55	3.20			2.63	3.27	1	3	4				0.00	0.36								
170	**trazodone**	0.48	0.15	0.20	0.23	0.76	1.75	0.78	0.80	0.26	0.26	3.72	4.24	0	6	6	3.41	-8.50	-6.21	-7.88	1.58	5.01	3.36	2.8	9.30	0.97	-0.66	1.00	0.52
171	**trimethoprim**	1.45	0.20	0.29	0.32	0.84	0.79	1.34	1.89	-0.24	-0.34	2.90	10.55	2	7	9	2.09	-8.80	-1.22	-8.68	0.58	4.14	2.63	-2.47					
172	**tropicamid**	0.72	0.20	0.29	0.30	0.83	1.55	1.91	1.84	-86	-83.00	2.84	5.34	1	3	4	4.06	-9.68	-2.81	-9.40	1.15	4.15	2.76	-0.01	1.06	0.03	-1.70	0.37	0.01
173	**valproic acid**	0.25	0.01	0.03	0.01	0.04	0.11	0.79	0.05	0.26	0.02	1.44	3.73	1	2	3	1.93	-11.19	10.01	-12.19	0.16	3.75	5.54	2.61	0.08	-1.09	-2.66	-1.02	-1.51
174	**valsartan**	19.40	0.18	0.23	0.25	0.97	0.86	0.95	1.01	0.23	0.25	4.22	11.21	2	8	10	2.92	-9.69	-6.39	-9.05	0.05	5.46	3.92	3.88	0.08	-1.10	-3.15	-1.08	-1.68
175	**venlafaxine**	0.57	0.14	0.22	0.21	0.61	1.99	1.37	2.41	0.26	0.45	2.77	3.27	1	3	4	3.25	-9.02	2.73	-9.29	1.07	4.44	2.85	1.44	8.25	0.92	-0.60	1.49	0.76
176	**verapamil**	0.37	0.11	0.19	0.19	0.86	1.34	0.58	0.96	0.92	1.51	4.55	6.40	0	6	6	5.44	-8.76	0.90	-8.85	2.33	7.51	4.53	0.57	11.97	1.08	-0.73	1.12	1.32
177	**warfarin**	-0.97	0.22	0.28	0.32	0.87	1.31	1.00	0.84	2.15	1.80	3.08	6.36	1	3	4	4.12	-7.38	-13.35	-6.05	-0.9	4.01	2.76	0.46					
178	**zolmitriptane**	-1.95	0.18	0.30	0.31	0.86	1.50	1.16	3.44	-0.29	-0.85	2.89	5.74	2	5	7	4.09	-8.73	-1.57	-8.57	-0.44	4.68	2.74	-1.01	3.30	0.52	-1.24	1.12	-0.51
179	**zolpidem**	0.25	0.23	0.25	0.26	0.76	2.05	0.96	0.84	1.02	0.90	3.07	3.76	0	4	4	6.69	-8.37	-0.45	-8.33	3.02	3.33	2.94	0.86	2.60	0.42	-1.16	0.45	0.48
180	**zopiclone**	-0.02	0.25	0.22	0.22	0.84	0.90	2.13	1.84	0.42	0.36	3.89	9.18	0	9	9	5.47	-9.13	-1.78	-8.95	-42	3.36	3.86	2.31	0.91	-0.04	-2.00	0.31	-0.40
181	**zuclopenthixol**	0.12	0.11	0.16	0.17	0.62	1.15	0.48	0.61	0.09	0.12	3.97	5.20	1	3	4	2.45	-7.57	-4.53	-7.12	5.06	5.58	3.55	4.97	48.73	1.69	-0.03	1.69	0.99
