# Supplementary material for: Chromatographic Data in Statistical Analysis of BBB Permeability Indices
Source: Membranes (Basel). 2023 Jun 26;13(7):623. doi: 10.3390/membranes13070623 (PMC10384010; doi:10.3390/membranes13070623)

Table S2. The structures of 181 APIs

1. acebutolol	2. aceclofenac	3. acenocumarol	4. acetazolamid
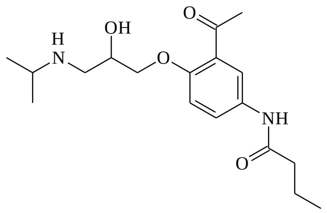	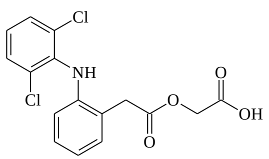	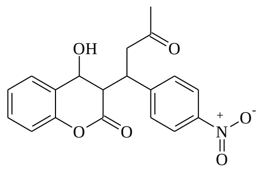	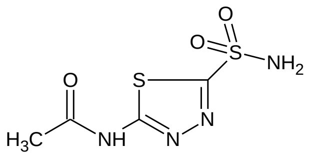
5. acetylsalicylic acid	6. acyclovir	7. allopurinol	8. alprazolam
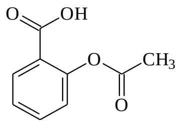	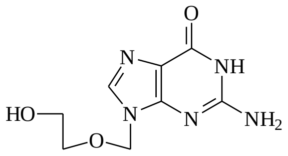	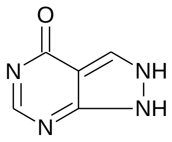	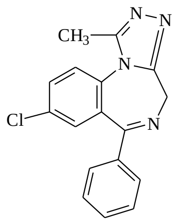
9. amiodarone	10. amitriptyline	11. amlodipine	12. amoxilcillin
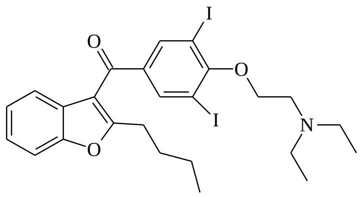	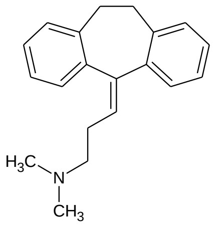	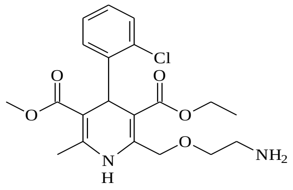	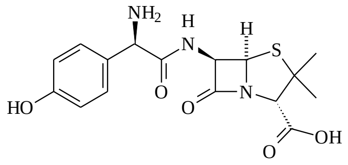
13. astemizol	14. atenolol	15. atorvastatin	16. atropine
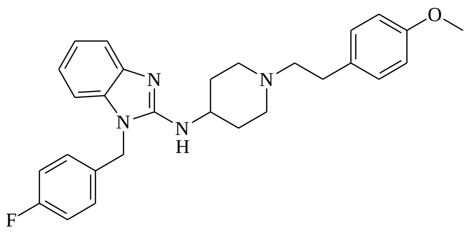	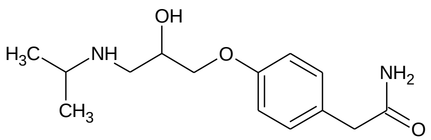	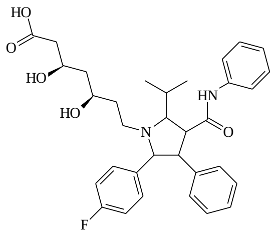	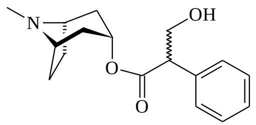

17. azithromycin

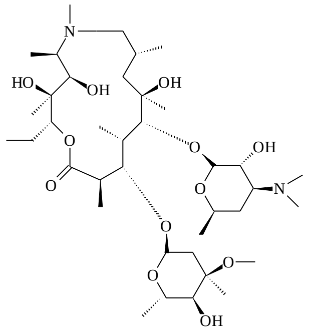

18. betahistine

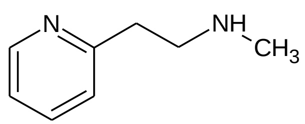

19. betaxolol

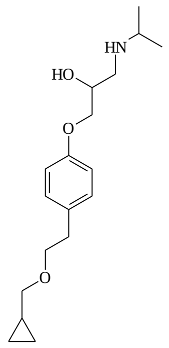

20. bilastine

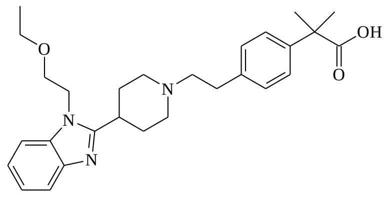

21. biperiden

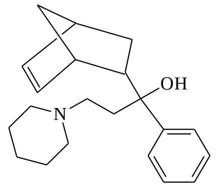

22. bisoprolol

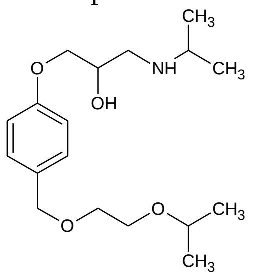

23. bromazepam

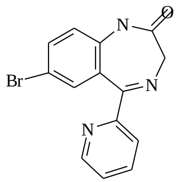

24. bromocriptine

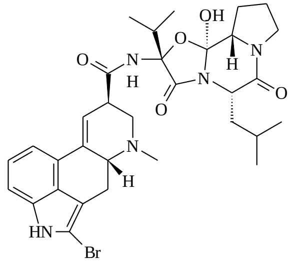

25. bupivacaine

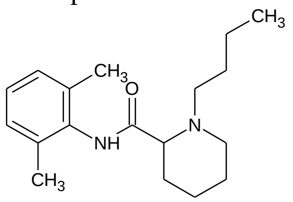

26. buspirone

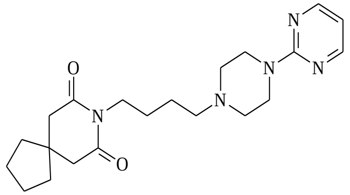

27. caffeine

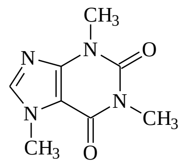

28. capecitabine

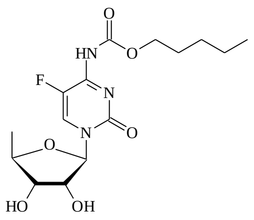

29. captopril

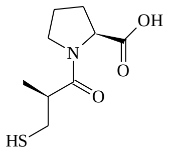

30. carbamazepine

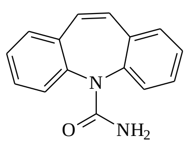

31. carbegoline

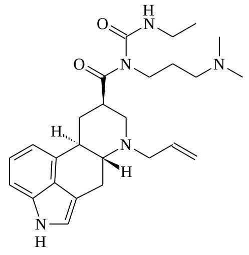

32. carvedilol

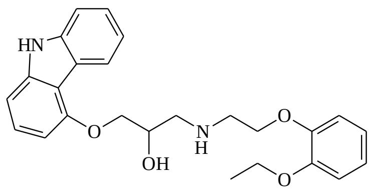

33. cefuroxime

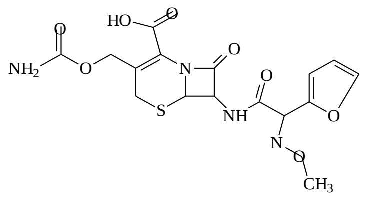

34. celecoxib

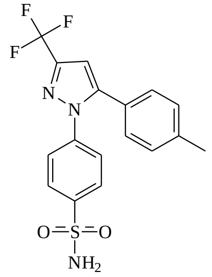

35. celiprolol

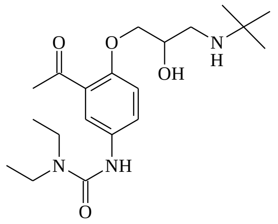

36. cephalexin

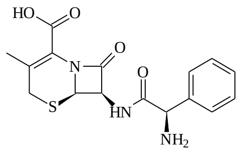

37. cetrizine

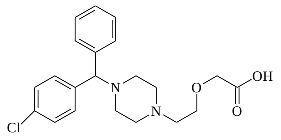

38. chloramphenicol

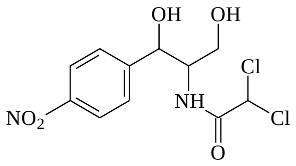

39. chloroquine

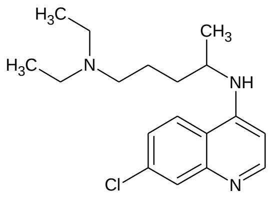

40. chlorpromazine

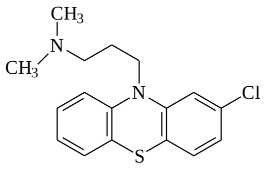

41. chlortalidone

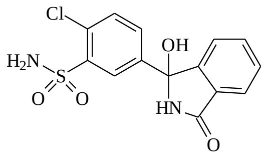

42. cimetidine

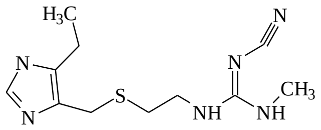

43. ciprofloxacin

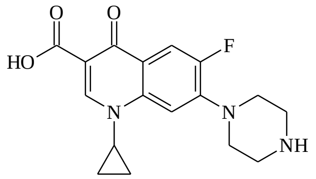

44. cisapride

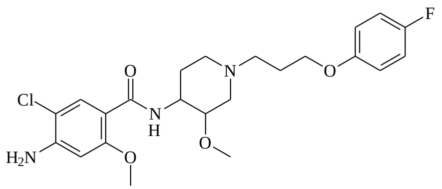

45. citalopram

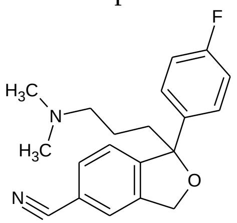

46. clarithromycin

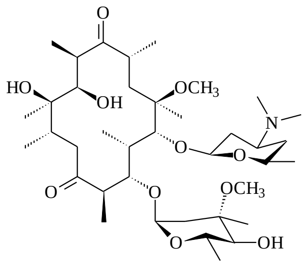

47. clindamycin

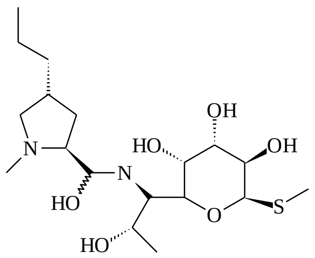

48. clobazam

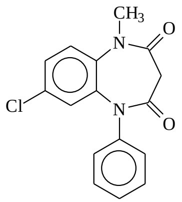

49. clomipramine

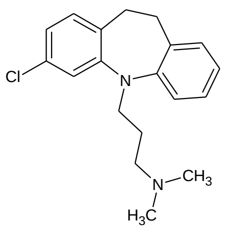

50. clonidine

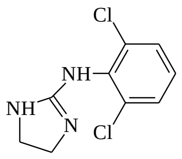

51. clorazepate

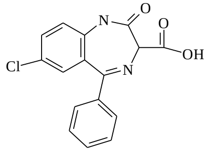

52. clozapine

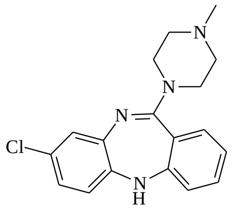

53. colchicine

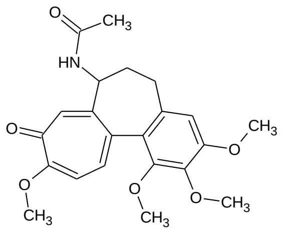

54. cyproheptadine

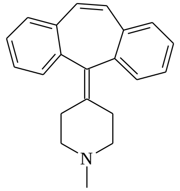

55. desloratidine

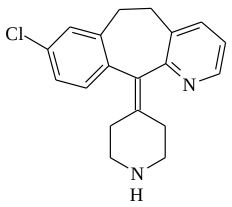

56. diazepam

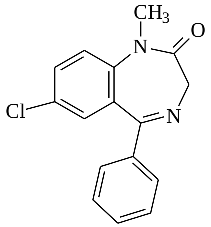

57. digoxin

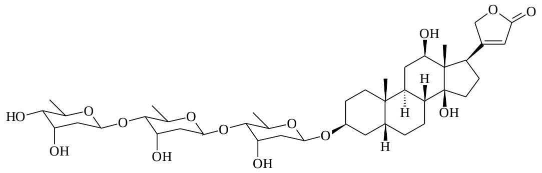

58. dihydroergotamine

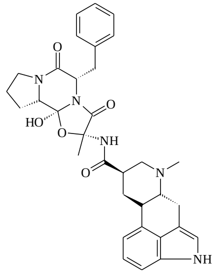

59. diltiazem

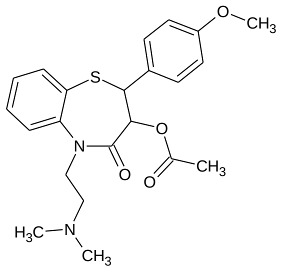

60. diphenhydramin

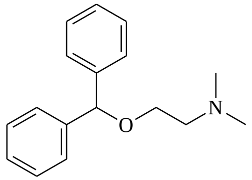

61. doxazosin

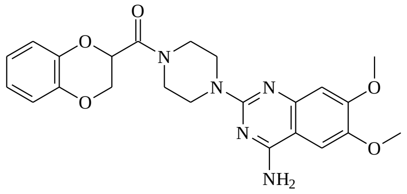

62. doxepin

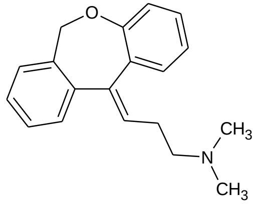

63. doxycycline

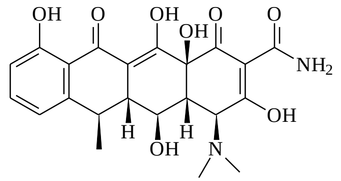

64. drotaverine

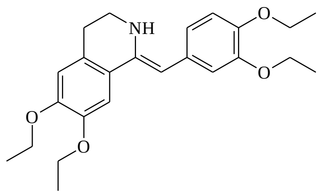

65. duloxetine

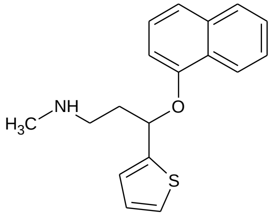

66. eletriptan

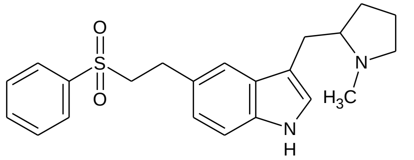

67.enalapril

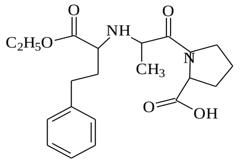

68. eplerenone

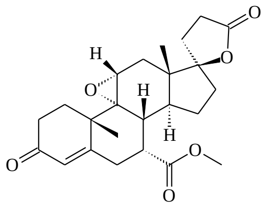

69. escitalopram

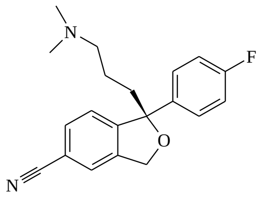

70. estradiol benzoate

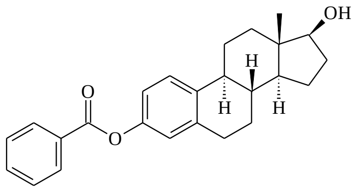

71. estrone

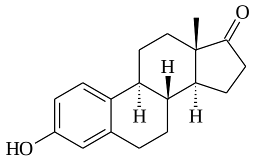

72. ethambutol

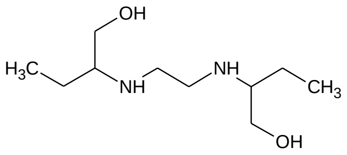

73. ethanol

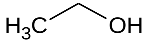

74. famotidine

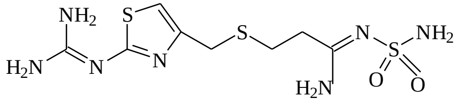

75. fexofenadine

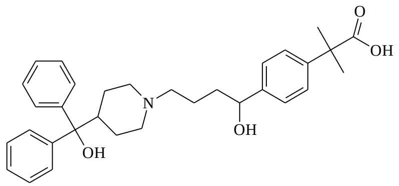

76. fluconazole

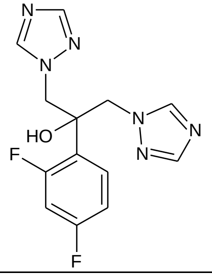

77. fluoxetine

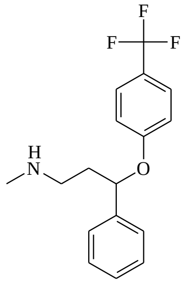

78. flupenthixol

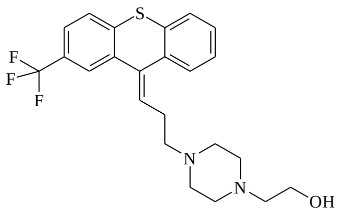

79. fluvoxamine

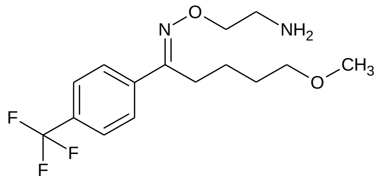

80. furosemide

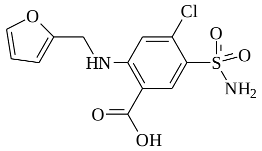

81. gabapentin

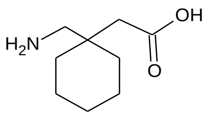

82. gentamycin

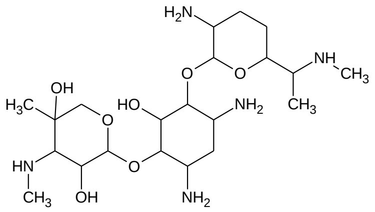

83. gliclazide

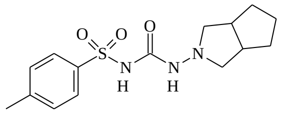

84. haloperidol

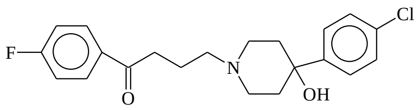

85. hydrochlorotiazide

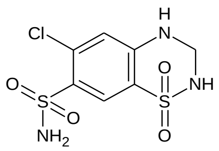

86. hydrocortisone acetate

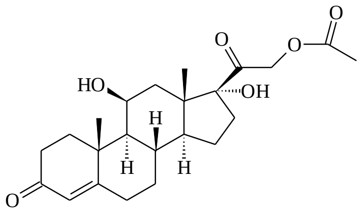

87. hydroxyzine

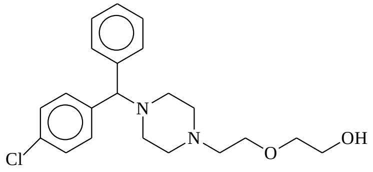

88. ibuprofen

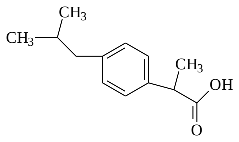

89. indomethacin

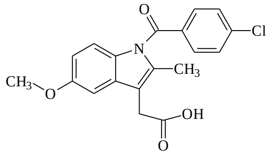

90. ipratropium

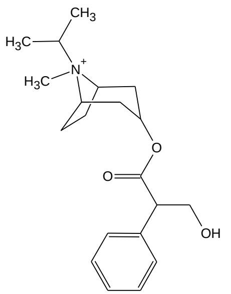

91. isosorbide

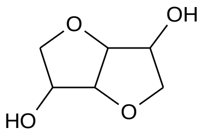

92. itraconazole

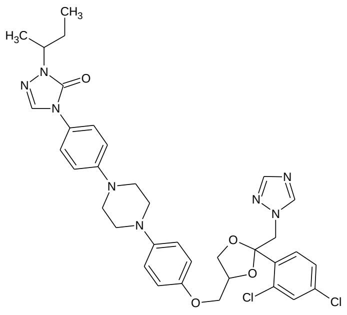

93. ketoprofen

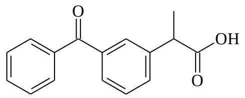

94. ketorolac

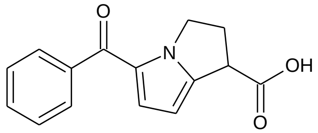

95. ketotifen

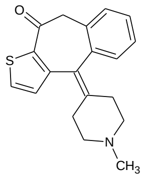

96. lamotrigine

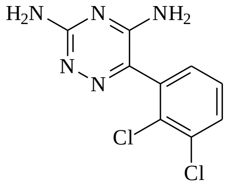

97. levetiracetam

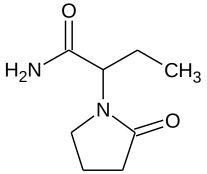

98. levocetrizine

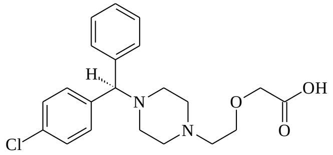

99. levofloxacin

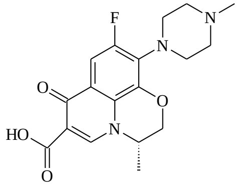

100. lincomycin

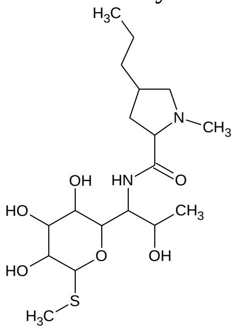

101. loperamide

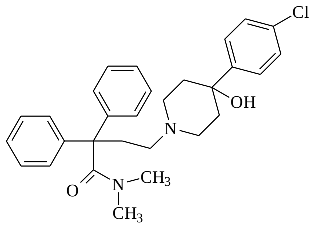

102. loratadine

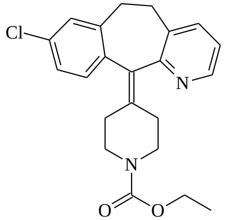

103. lorazepam

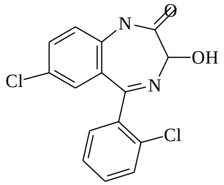

104. medazepam

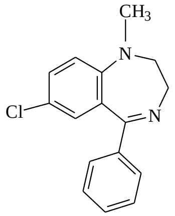

105. meloxicam

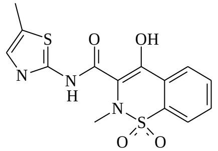

106. mesalazine

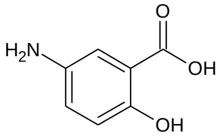

107. metformin

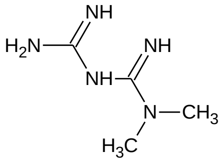

108. methyl paraben

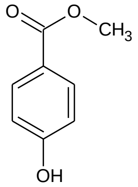

109. methyldopa

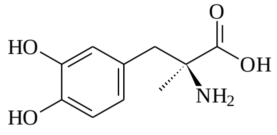

110. metoclopramide

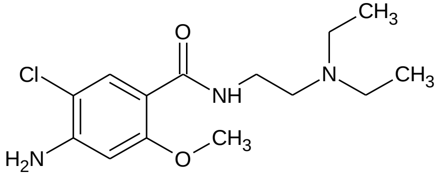

111. metoprolol

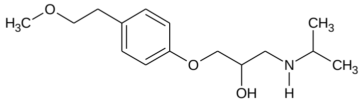

112. mianserin

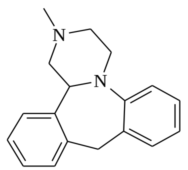

113. midazolam

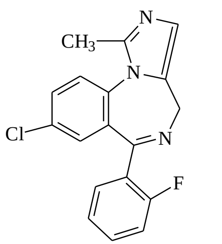

114. minoxidil

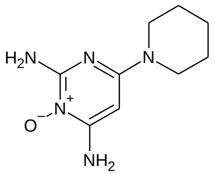

115. mirtazapine

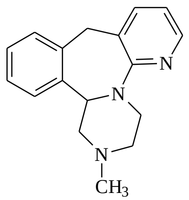

116. montelukast

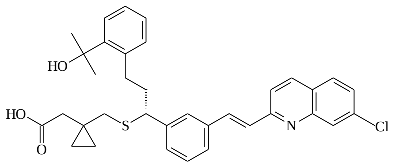

117. naproxen

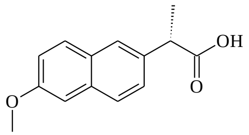

118. nebivolol

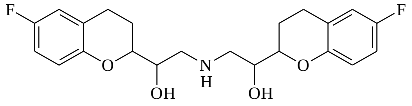

119. nitrendipine

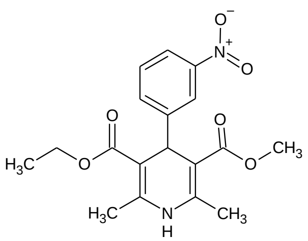

120. ofloxacin

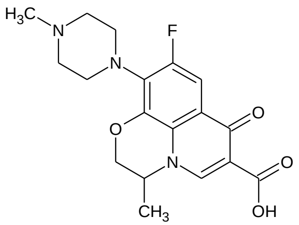

121. olanzapine

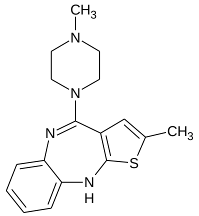

122. oxazepam

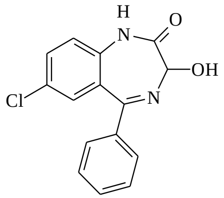

123. oxcarbamazepine

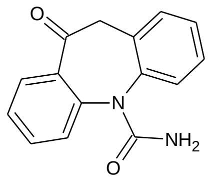

124. oxybutynin

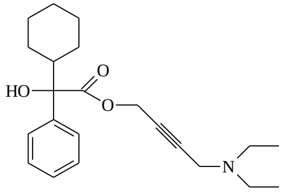

125. PABA

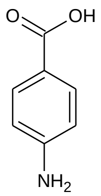

126. pantoprazole

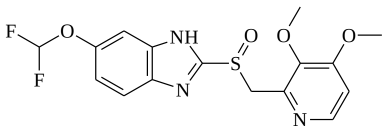

127. paracetamol

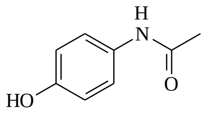

128. paroxetine

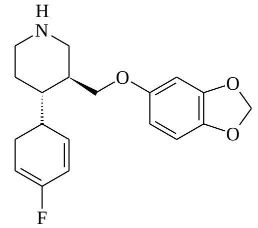

129. pefloxacin

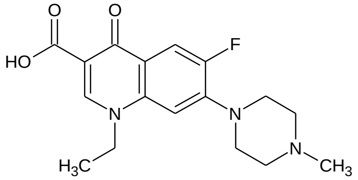

130. pergolide

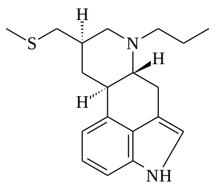

131. perindopril

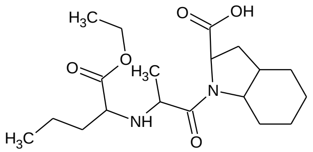

132. phenytoin

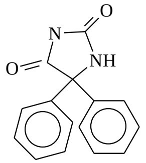

133. pindolol

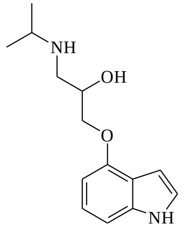

134. piroxicam

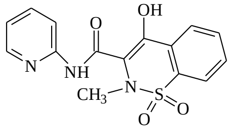

135. prednizolone

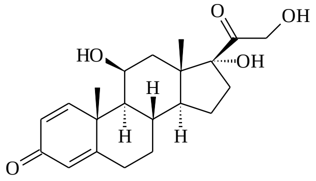

136. pregabalin

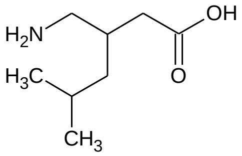

137. primidone

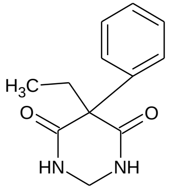

138. progesterone

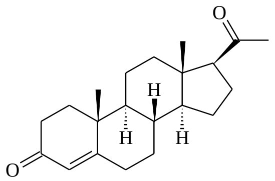

139. promazine

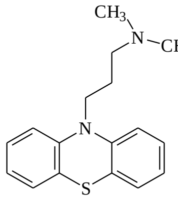

140. promethazine

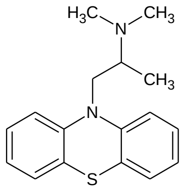

141. propafenone

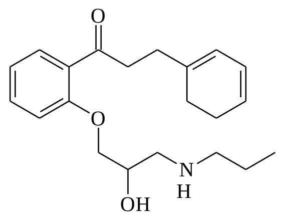

142. propranolol

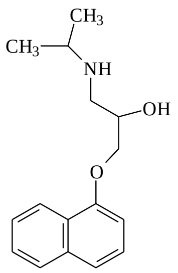

143. propylthiouracil

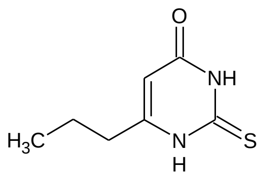

144. pseudoephedrine

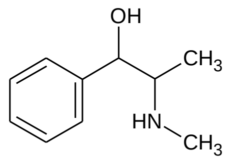

145. quetiapine

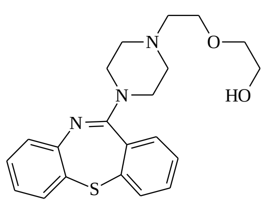

146. quinapril

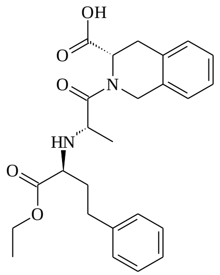

147. quinine

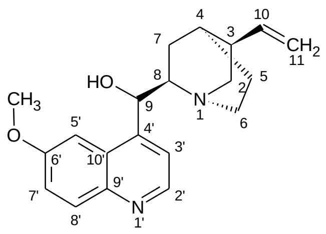

148. ranitidine

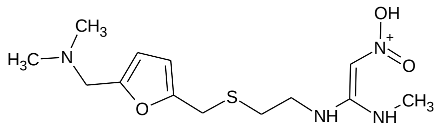

149. rifampicin

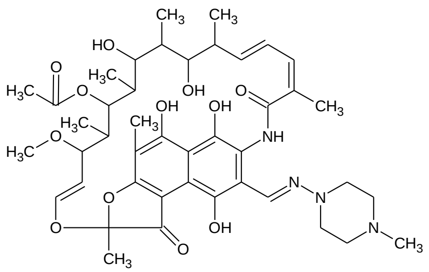

150. rimantadine

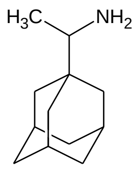

151. risperidone

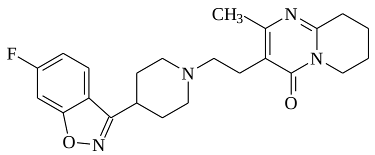

152. rizatriptan

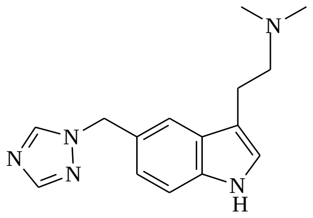

153. rosuvastatin

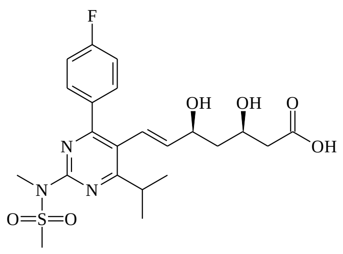

154. roxitromicin

155. rupatadine

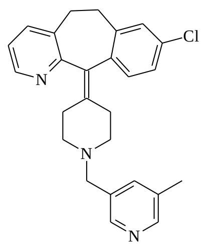

156. sertraline

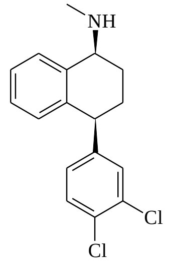

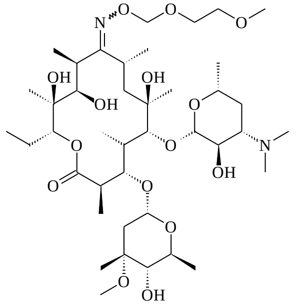

157. sildenafil

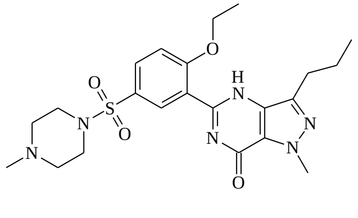

158. simvastatin

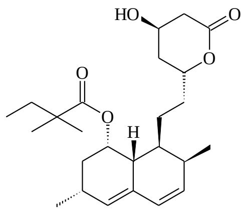

159. sotalol

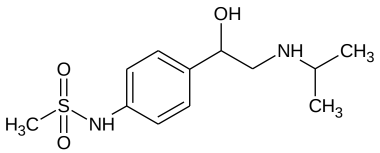

160. spironolactone

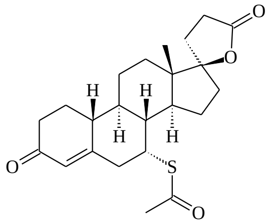

161. sulpiride

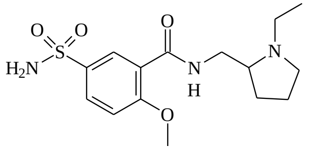

162. telmisartan

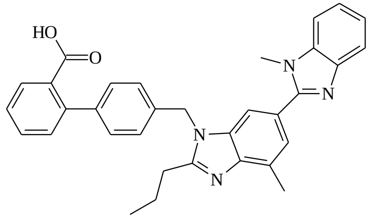

163. temazepam

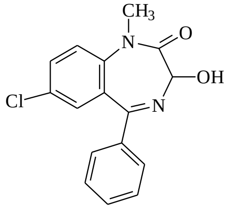

164. theophylline

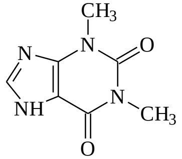

165. thioridazine

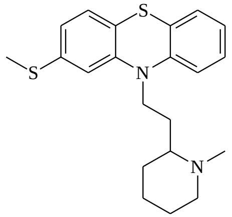

166. timolol

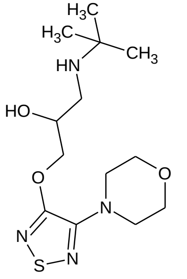

167. tinidazole

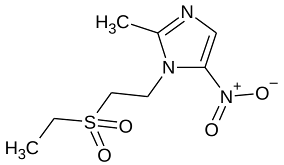

168. tolterodine

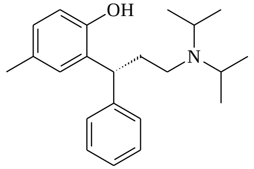

169.tramadol

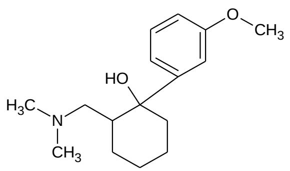

170. trazodone

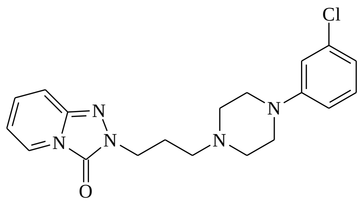

171. trimethoprim

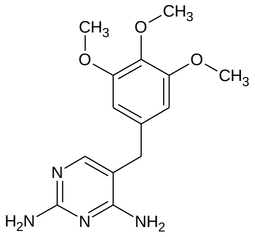

172. tropicamid

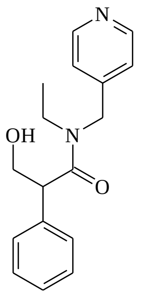

173. valproic acid

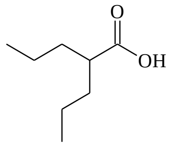

174. valsartan

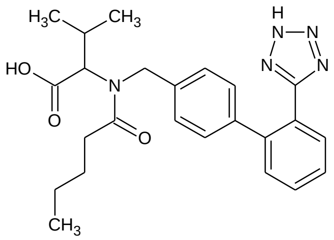

175. venlafaxine

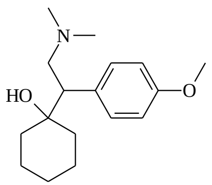

176. verapamil

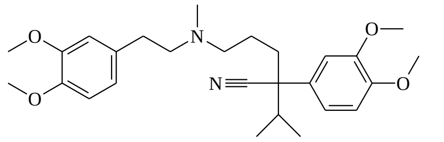

177. warfarin

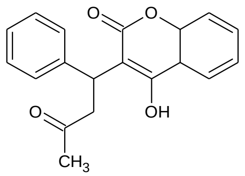

178. zolmitriptane

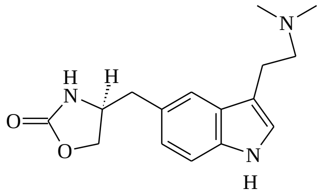

179. zolpidem

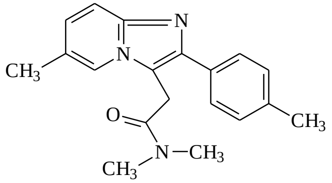

180. zopiclone

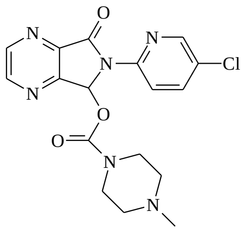

181. zuclopenthixol

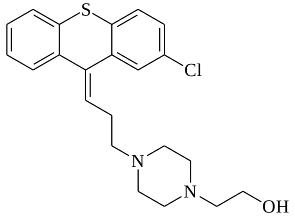

Supplement: Supplementary file 1 [file membranes-13-00623-s001.zip › membranes-2455267-supplementary/Wanat Supplementary material/Wanat Table S2. Structures of 181 APIs.pdf]
