# Supplementary material for: Chromatographic Data in Statistical Analysis of BBB Permeability Indices
Source: Membranes (Basel). 2023 Jun 26;13(7):623. doi: 10.3390/membranes13070623 (PMC10384010; doi:10.3390/membranes13070623)
Supplement: Supplementary file 1 [file membranes-13-00623-s001.zip › membranes-2455267-supplementary/Wanat Supplementary material/Wanat Table S3. Corelation matrix of molecular descriptors.pdf]

Table S3. Correlation matrix of molecular descriptors

	mean	SD	**B1**	**B2**	**B2>-0.9**	**B2>-0.52**	**CNS+/-**	**$K_{p.uu.brain}$**	**PhCharg**	**pKa**	**logU/D**	**PB**	**NP**	**RP**	**MW**	**PSA**	**HD**	**HA**	**HA+D**	**DM**	**eH**	**eL**	**eH-eL**	**logD**	**Sa**	**V**	**logP**	**logkIAM**
**B1**	-0.490	0.947	1.000	0.519	0.378	0.335	0.194	0.301	0.231	0.070	0.182	0.277	-0.147	-0.370	0.054	-0.516	-0.467	-0.389	-0.484	-0.166	0.026	0.208	-0.030	0.190	0.106	0.127	0.315	0.397
**B2**	-0.605	0.692	0.519	1.000	0.780	0.750	0.570	0.274	0.075	0.166	0.034	0.204	-0.257	-0.294	-0.036	-0.998	-0.765	-0.850	-0.927	-0.228	0.047	0.176	-0.292	0.209	-0.221	-0.252	0.213	0.390
**B2>-0.9**	0.729	0.446	0.378	0.780	1.000	0.692	0.550	0.180	0.137	0.197	0.019	0.081	-0.213	-0.211	0.044	-0.773	-0.519	-0.653	-0.681	-0.200	0.115	0.131	-0.177	0.225	-0.146	-0.194	0.136	0.326
**B2>-0.52**	0.564	0.497	0.335	0.750	0.692	1.000	0.521	-0.044	0.171	0.157	-0.025	0.129	-0.130	-0.187	-0.128	-0.744	-0.499	-0.653	-0.673	-0.189	0.090	0.176	-0.139	0.205	-0.228	-0.221	0.177	0.277
**CNS+/-**	0.719	0.451	0.194	0.570	0.550	0.521	1.000	0.178	0.070	0.225	0.035	0.202	-0.073	-0.064	0.008	-0.570	-0.518	-0.548	-0.608	-0.218	0.010	0.018	-0.175	0.229	-0.279	-0.282	0.164	0.263
**$K_{u,pp,brain}$**	1.880	1.468	0.301	0.274	0.180	-0.044	0.178	1.000	0.142	-0.088	0.124	0.237	-0.189	-0.519	-0.026	-0.274	-0.155	-0.259	-0.239	-0.089	0.276	0.042	0.207	0.174	0.047	-0.020	0.312	0.474
**PhCharg**	0.354	0.859	0.231	0.075	0.137	0.171	0.070	0.142	1.000	0.548	0.312	-0.148	-0.428	-0.302	0.028	-0.073	0.060	0.011	0.035	-0.146	0.162	0.197	0.122	0.056	0.177	0.053	-0.093	0.423
**pKa**	7.613	2.757	0.070	0.166	0.197	0.157	0.225	-0.088	0.548	1.000	0.009	-0.022	-0.269	-0.232	0.043	-0.166	-0.059	-0.157	-0.134	-0.193	-0.028	0.167	-0.020	0.108	-0.014	-0.034	0.012	0.353
**logU/D**	-2.324	1.530	0.182	0.034	0.019	-0.025	0.035	0.124	0.312	0.009	1.000	0.146	-0.131	-0.246	0.048	-0.034	-0.053	0.043	0.006	-0.003	0.274	0.006	0.179	0.028	0.178	0.133	0.004	0.249
**PB**	0.722	0.300	0.277	0.204	0.081	0.129	0.202	0.237	-0.148	-0.022	0.146	1.000	0.200	-0.190	-0.101	-0.208	-0.303	-0.183	-0.261	-0.037	0.141	-0.121	0.042	0.355	0.107	0.256	0.439	0.364
**NP**	0.662	0.265	-0.147	-0.257	-0.213	-0.130	-0.073	-0.189	-0.428	-0.269	-0.131	0.200	1.000	0.539	-0.190	0.251	0.119	0.161	0.164	0.000	-0.157	-0.047	-0.019	-0.039	-0.204	-0.025	0.128	-0.124
**RP**	0.723	0.194	-0.370	-0.294	-0.211	-0.187	-0.064	-0.519	-0.302	-0.232	-0.246	-0.190	0.539	1.000	-0.124	0.297	0.193	0.202	0.225	0.020	-0.076	-0.057	-0.131	-0.190	-0.167	-0.217	-0.132	-0.340
**MW**	5.912	26.390	0.054	-0.036	0.044	-0.128	0.008	-0.026	0.028	0.043	0.048	-0.101	-0.190	-0.124	1.000	0.036	0.057	0.069	0.074	0.079	0.031	-0.014	0.021	-0.060	-0.031	-0.029	-0.103	-0.054
**PSA**	7.178	4.318	-0.516	-0.998	-0.773	-0.744	-0.570	-0.274	-0.073	-0.166	-0.034	-0.208	0.251	0.297	0.036	1.000	0.771	0.852	0.932	0.222	-0.038	-0.178	0.298	-0.211	0.220	0.249	-0.221	-0.394
**HD**	1.757	1.728	-0.467	-0.765	-0.519	-0.499	-0.518	-0.155	0.060	-0.059	-0.053	-0.303	0.119	0.193	0.057	0.771	1.000	0.530	0.815	0.036	0.024	0.020	0.171	-0.176	0.043	0.052	-0.305	-0.308
**HA**	4.801	2.611	-0.389	-0.850	-0.653	-0.653	-0.548	-0.259	0.011	-0.157	0.043	-0.183	0.161	0.202	0.069	0.852	0.530	1.000	0.923	0.260	0.003	-0.161	0.384	-0.230	0.361	0.402	-0.099	-0.357
**HA+D**	6.558	3.819	-0.484	-0.927	-0.681	-0.673	-0.608	-0.239	0.035	-0.134	0.006	-0.261	0.164	0.225	0.074	0.932	0.815	0.923	1.000	0.190	0.014	-0.096	0.341	-0.237	0.260	0.292	-0.209	-0.390
**DM**	3.673	2.362	-0.166	-0.228	-0.200	-0.189	-0.218	-0.089	-0.146	-0.193	-0.003	-0.037	0.000	0.020	0.079	0.222	0.036	0.260	0.190	1.000	-0.007	-0.316	0.228	-0.132	-0.026	0.041	-0.119	-0.119
**eH**	-9.013	0.700	0.026	0.047	0.115	0.090	0.010	0.276	0.162	-0.028	0.274	0.141	-0.157	-0.076	0.031	-0.038	0.024	0.003	0.014	-0.007	1.000	0.042	0.678	0.157	0.117	0.131	0.056	0.280
**eL**	-3.300	7.268	0.208	0.176	0.131	0.176	0.018	0.042	0.197	0.167	0.006	-0.121	-0.047	-0.057	-0.014	-0.178	0.020	-0.161	-0.096	-0.316	0.042	1.000	-0.706	-0.095	-0.157	-0.115	0.028	-0.041
**eH-eL**	-8.440	1.734	-0.030	-0.292	-0.177	-0.139	-0.175	0.207	0.122	-0.020	0.179	0.042	-0.019	-0.131	0.021	0.298	0.171	0.384	0.341	0.228	0.678	-0.706	1.000	0.135	0.198	0.177	0.094	0.030
**logD**	0.883	3.904	0.190	0.209	0.225	0.205	0.229	0.174	0.056	0.108	0.028	0.355	-0.039	-0.190	-0.060	-0.211	-0.176	-0.230	-0.237	-0.132	0.157	-0.095	0.135	1.000	0.189	0.161	0.220	0.361
**Sa**	4.550	1.263	0.106	-0.221	-0.146	-0.228	-0.279	0.047	0.177	-0.014	0.178	0.107	-0.204	-0.167	-0.031	0.220	0.043	0.361	0.260	-0.026	0.117	-0.157	0.198	0.189	1.000	0.772	0.147	0.337
**V**	3.021	0.956	0.127	-0.252	-0.194	-0.221	-0.282	-0.020	0.053	-0.034	0.133	0.256	-0.025	-0.217	-0.029	0.249	0.052	0.402	0.292	0.041	0.131	-0.115	0.177	0.161	0.772	1.000	0.350	0.361
**logP**	1.150	2.027	0.315	0.213	0.136	0.177	0.164	0.312	-0.093	0.012	0.004	0.439	0.128	-0.132	-0.103	-0.221	-0.305	-0.099	-0.209	-0.119	0.056	0.028	0.094	0.220	0.147	0.350	1.000	0.226
**logkIAM**	0.450	0.923	0.397	0.390	0.326	0.277	0.263	0.474	0.423	0.353	0.249	0.364	-0.124	-0.340	-0.054	-0.394	-0.308	-0.357	-0.390	-0.119	0.280	-0.041	0.030	0.361	0.337	0.361	0.226	1.000
